# Environmental Yeast Abundance and Diversity Assessment in Recreation Areas of Bangkok, Thailand

**DOI:** 10.1111/1758-2229.70212

**Published:** 2025-10-21

**Authors:** Pantira Singkum, Thanwa Wongsuk, Potjaman Pumeesat, Rattiya Cheewapat, Ingo Ebersberger, Rapee Thummeepak, Amornrat Aroonnual

**Affiliations:** ^1^ Department of Microbiology and Immunology, Faculty of Tropical Medicine Mahidol University Bangkok Thailand; ^2^ Department of Clinical Pathology, Faculty of Medicine, Vajira Hospital Navamindradhiraj University Bangkok Thailand; ^3^ Department of Medical Technology, Faculty of Science and Technology Bansomdejchaopraya Rajabhat University Bangkok Thailand; ^4^ Department of Microbiology and Parasitology, Faculty of Medical Science Naresuan University Phitsanulok Thailand; ^5^ Applied Bioinformatics Group, Institute of Cell Biology and Neuroscience, Department of Biosciences Goethe University Frankfurt am Main Germany; ^6^ Senckenberg Biodiversity and Climate Research Centre (S‐BIKF) Frankfurt am Main Germany; ^7^ LOEWE Centre for Translational Biodiversity Genomics (TBG) Frankfurt am Main Germany; ^8^ Department of Tropical Nutrition and Food Science, Faculty of Tropical Medicine Mahidol University Bangkok Thailand

**Keywords:** environmental yeasts, human activity, ITS region, yeast diversity, yeast identification

## Abstract

The diversity of environmental yeast communities is underestimated in tropical and sub‐tropical regions. Numerous studies demonstrated that human activity can alter the yeast diversity and increase pathogenic yeast proportions, indicating that people who frequently visit those areas are at risk of being infected. The main purpose of this study was to investigate the abundance and diversity of yeasts obtained from recreation areas in Bangkok. In this study, 158 soil and water samples were collected from 12 public parks. The analysis of the yeast communities revealed different patterns among recreation areas. Moreover, we aimed to identify yeasts using the internal transcribed spacer (ITS) region. Yeast isolates were identified into 22 genera, with *Candida* being the most common. Although the ITS region may be used to distinguish yeasts at the genus level, some isolates remain unidentified. Thus, our findings are the first report highlighting the diversity of yeast from recreation areas in Bangkok. Our study also provides information on the ITS region for environmental yeast identification, suggesting that this region might be appropriate for some yeast taxa. In conclusion, this study proposes that the abundance and diversity of yeast may differ due to several factors, such as the surrounding environment, park landscapes, and water supplies for park maintenance.

## Graphical abstract

Isolation and identification of yeasts isolated from 12 recreation areas in Bangkok, Thailand. Yeast isolates were identified using conventional methods and molecular techniques. The abundance and diversity of yeast isolates were assessed using the internal transcribed spacer region.

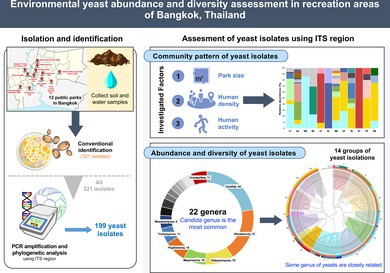

## 1 Introduction

Over the past two decades, biodiversity has seen an explosion of interest, especially in environmental sources. Soil and water are very heterogeneous habitats containing a great diversity of microorganisms (Frac et al. 2018). In recent years, up to 340 species of yeast and yeast‐like fungi have been found in natural environments worldwide (Coleman et al. 2018; Miceli et al. 2011). Several yeasts have been typically isolated from environmental sources such as freshwater, ocean, brackish water, soil, plants, and animals (Moazeni et al. 2022). Most studies on yeast diversity have been reported from tropical and subtropical regions (Boekhout et al. 2021; Rosa et al. 2023). In Thailand, the diversity of yeasts has been assessed in a variety of natural resources, including mangroves, forests, farms, oceans, waterfalls, and caves (Rosa et al. 2023; Into et al. 2020; Kaewkrajay et al. 2020; Kaewwichian and Khamthaiklang 2022; Kanpiengjai et al. 2023; Nualmalang et al. 2023; Sapsirisuk et al. 2022; Satianpakiranakorn et al. 2020). Numerous currently known yeasts are important for environmental, economic, and/or medical relevance. However, some groups may become potential human and animal pathogens and pose serious problems to human health.

The available evidence confirms the hypothesis that opportunistic fungal diseases are re‐emerging worldwide from natural sources and causing severe disease in humans, especially in the immunocompromised populations (Lass‐Florl and Steixner 2023). Yeasts potentially adapt to survive in a variety of environmental conditions. Environmental yeasts exhibit strong endemism, as well as a remarkable number of currently undiscovered species. However, like other environmental dwellers, yeasts are often threatened by habitat changes caused by human activities, for example, agriculture, forestry, deforestation, and urbanisation (Mok et al. 1984; Slavikova and Vadkertiova 2003; Yurkov 2018). Several studies investigated yeast populations in different natural sources (including recreational facilities, playgrounds, parks, and gardens) that are connected to many human activities (Wojcik et al. 2013; Moazeni et al. 2022; Biedunkiewicz and Góralska 2016; Biedunkiewicz et al. 2020; Brandão et al. 2021). They found that the yeast distribution in the environment was mostly demonstrated in polluted and agricultural areas. They suggested that the yeast diversity is significantly influenced by specific locations and their surrounding conditions (Ayanbimpe et al. 2013; Monapathi et al. 2017; Montanari et al. 2018; Silva‐Bedoya et al. 2014). Additionally, previous studies have indicated that highly polluted areas can serve as habitats for many yeast species, including *Rhodotorula, Candida*, and *Cryptococcus*. These findings also revealed that yeast species are associated with contamination resulting from human activities (Biedunkiewicz and Góralska 2016; Biedunkiewicz et al. 2020; Brandão et al. 2021).

The interactions between soil, water, and humans are critical to human health and well‐being. Some studies indicated that the ratio of yeast in recreation areas is linked with human activities. Yeasts can exist in the environment via the emission of contaminated wastes from human communities. This suggests that populations who frequently use public parks and engage in outdoor activities are particularly at risk of being infected (Wojcik et al. 2013; Glushakova et al. 2022; Monapathi et al. 2020). However, the risk of yeast infection from contact with these materials in recreation areas is still unclear, depending on the individual's immune system (Sakshi and Alka 2015; Goralska et al. 2020).

Nowadays, several approaches, such as morphological, biochemical tests, as well as molecular tools, are used as yeast identification techniques. Normally, the accurate method for yeast identification largely relies on molecular techniques. The evaluation of specific nucleotide sequences can be a precise method for resolving the taxonomic issues (Abdel‐Sater et al. 2016; Vetrovsky et al. 2020; Wu et al. 2019). In this regard, many ribosomal genes are particularly representative of the consistent evaluative markers, with alternating conserved regions and variable regions (Banos et al. 2018; Asemaninejad et al. 2016; Collins and Cruickshank 2013; Edwards et al. 2017; Raja et al. 2017). The most widely used molecular targets for yeast are the 28S nuclear ribosomal DNA (nrDNA) and the internal transcribed spac (ITS) region, which includes the ITS1–5.8S nrDNA–ITS2 gene cluster (Badotti et al. 2017; Batovska et al. 2017; Fujita et al. 2001; Op De Beeck et al. 2014; Schoch et al. 2012). A previous study suggested that these regions should be used to identify taxa and analyse phylogenetic relatedness of yeasts (Nilsson et al. 2015). Although using the ITS region has been possible to recognise the yeast taxa, the accuracy of this region seems to be obstructed by strain variability and closely related species. Moreover, the identification of some environmental yeast strains is still challenged depending on their sequence database (Zhao et al. 2018; Leaw et al. 2006).

In the last decade, the taxonomists concluded that yeast populations depend on external stimuli, including temperature, residence, and the moisture content (Yurkov 2018). However, there have been few studies on the diversity of yeast in soil and water resources in recreation areas, especially in Thailand (Aljohani et al. 2018; Dabassa Koricha et al. 2019; Into et al. 2020; Klaubauf et al. 2010; Samarasinghe et al. 2021). Due to the information on the association between yeasts and natural habitat being limited, we aim to investigate the yeast richness, abundance, and diversity in recreation areas of Bangkok, Thailand. Moreover, the association of yeast diversity with human activities was also studied. The yeast genera were identified using molecular techniques. The composition and abundance of the yeast community in the soil and water were assessed using genomic DNA analyses based on Internal transcribed spacer (ITS). The information from this study can fill the gap in knowledge in the environmental yeast communities of public parks in Thailand. In addition, this study also provides the information to verify and evaluate the ITS region to identify environmental yeasts.

## 2 Experimental Procedures

### 2.1 Study Area and Sample Collection

Samples were collected in Bangkok, the capital city of Thailand. The city is located in the tropical climate zone. The temperature varied between 25°C and 38°C, and the average humidity is 82% (Phanprasit et al. 2021). Currently, Bangkok has 5,527,994 inhabitants, with 2,809,300 temporary residents in 2021 (data published by the Department of City Planning and Urban Development, updated in 2021) and has 40 green areas in the form of large public parks, being places for human recreation.

**FIGURE 1:**
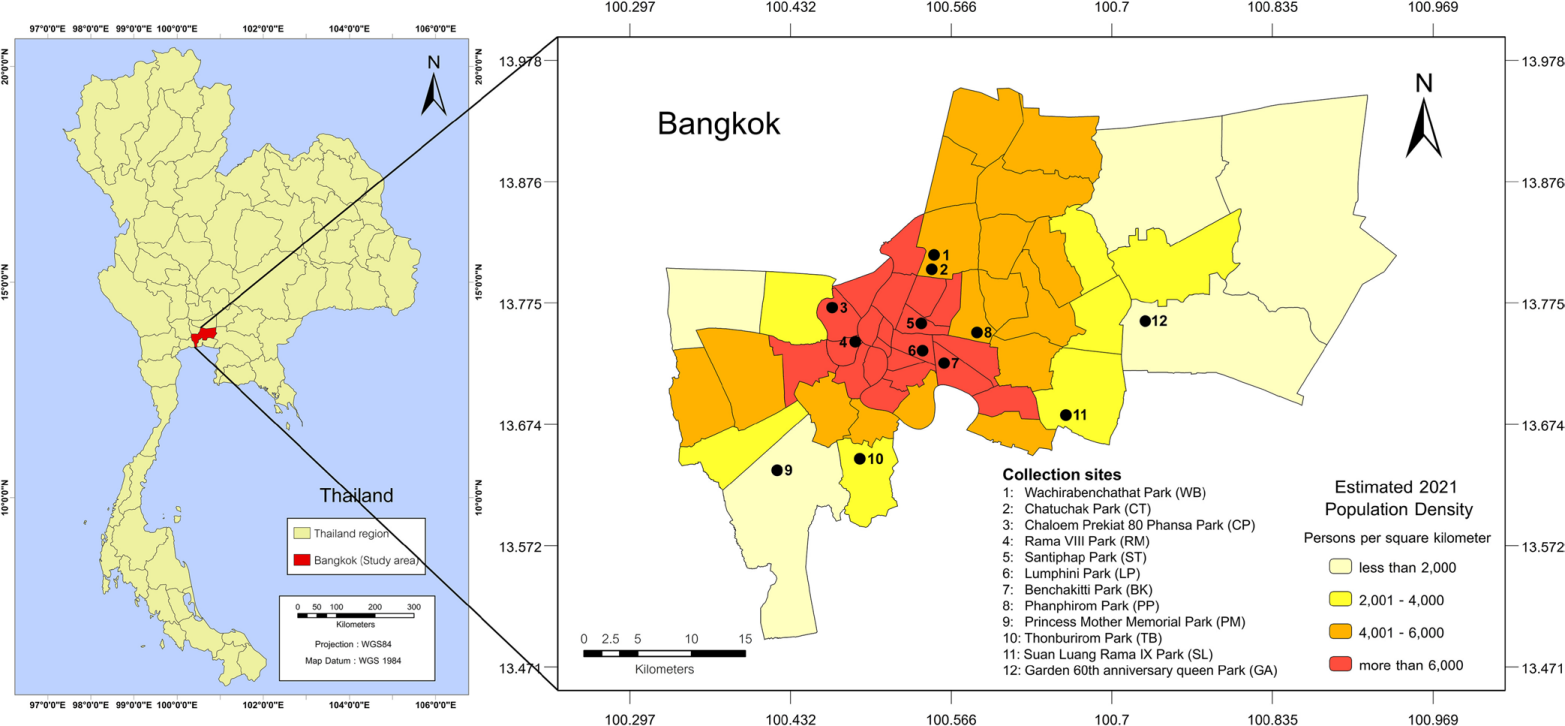
Map of the sampling sites and the geographical location of 12 recreation areas in Bangkok province, Thailand. The information on population density in Bangkok was provided by the Department of City Planning and Urban Development, updated in 2021. This map was created in ArcGIS 10.8.2 (http://www.esri.com/arcgis) using the georeferenced shapefiles obtained from the Bangkok Metropolitan Administration or BMA (https://data.bangkok.go.th/).

The study was conducted with permission from the Public Park Officer, Ministry of Environmental of Bangkok, Thailand. Samples were collected in the dry season during December 2019–February 2020 and December 2020–March 2021. The temperature during sampling periods varied between 26°C and 31°C, whereas the relative humidity varied between 60% RH and 75% RH. The data of temperature and humidity during sampling periods were derived from (Table S1). The 12 popular public parks located in urban areas with different ranges of human density were selected for sample collection, including Santiphap Park (ST), Lumphini Park (LP), Benchakitti Park (BK), Wachirabenchathat Park (WB), Suan Luang Rama IX Park (SL), Chaloem Prekiat 80 Phansa Park (CP), Chatuchak Park (CT), Rama VIII Park (RM), Phanphirom Park (PP), Princess Mother Memorial Park (PM), Thonburirom Park (TB), and Garden 60th Anniversary Queen Park (GA) (Figure 1). The Global Positioning System (GPS) coordinates of each site were determined using a Handy GPS application (Mobile version, Binary Earth) (Table S2).

The individual samples were distributed around the studied areas. Both soil and water samples were randomly collected from 10 sites in each park. The collection points were recorded as the GPS coordinates (Table S2). These GPS coordinates were constructed and the map of collection sites was plotted as shown in Figure S1. At each spot, the top layer of the soil was taken at a depth of 10–15 cm using a sterilised shovel. The surface of the soil was dug in a V‐shape, creating a 2 cm hole at the bottom. Soil samples were collected from the bottom, approximately 5–10 g of fine soil particles per sample using a sterilised metal spoon and were kept in a sterilised plastic bag. The water samples were collected at a depth of up to 30 cm from the surface. Surface water samples were taken in duplicate at each site using sterile 50 mL centrifuge tubes. Samples were placed on ice and transported to the laboratory. Samples were processed within 24 h of sample collection.

### 2.2 Isolation and Screening of Yeast Strains

**FIGURE 2:**
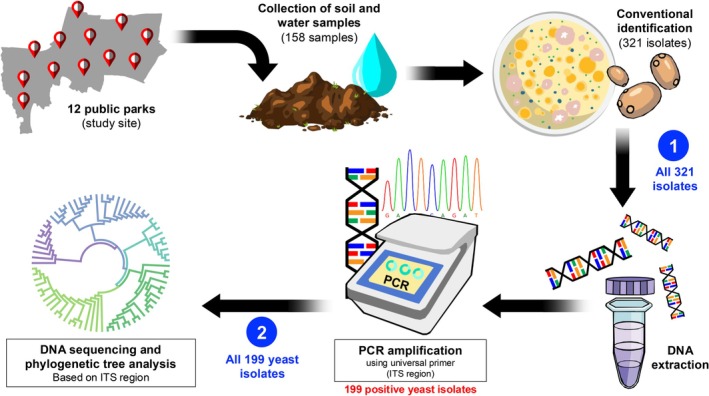
Scheme of sample collection and yeast identification. Soil and water samples (158 total) were collected from 12 public parks. Single isolates of 321 yeast‐like colonies were discovered with 243 from soil and 78 from water samples. Yeast isolates were further identified by molecular diagnostics using PCR technique. The results from the ITS region showed 199 out of 321 yeast‐like isolates. Then, the nucleotide sequences were generated for the phylogenetic tree.

The experimental schematic of this study was displayed in Figure 2. Yeast isolations were done following the protocol of Jamali and Gharaei (2015). Soil (0.2 g) was suspended into 10 mL of sterilised deionised water (1:50 soil/DW suspension). Soil solutions were allowed to stand for 10 min, followed by vortexing for 30 s. Then, 100 μL of each soil sample solution was spread on Sabouraud Dextrose Agar (SDA) (HiMedia, India) supplemented with 0.04 mg/mL chloramphenicol (Sigma‐Aldrich, USA) to inhibit bacterial growth. Water samples were mixed by vortex for 30 s. The water sample (100 μL) was directly spread on the SDA agar plate and incubated for 48 h at 25°C and 37°C. In order to isolate yeast colonies, the incubation period must not exceed 48 h because the moulds will overgrow and cover all of the yeast colonies, resulting in non‐purified yeast colony. Thus, in this experiment, we use an incubation period of not more than 2 days as a cutoff for yeast screening and isolation.

Samples were done in triplicate. The formation of yeast colonies was monitored daily for a week period, and their yeast characteristics were examined microscopically by Lactophenol Cotton Blue (LPCB) staining. To identify a yeast colony, they appear as smooth surface, raised colony, creamy texture, as well as various colours and shapes. To confirm each yeast isolate, colonies with a yeast‐like consistency were examined under the microscope by a wet mount technique. Yeast isolates were stained with Lactophenol Cotton Blue (LPCB) solution and observed under the microscope. The yeast cells are typically round or oval shapes, single or budding‐celled with blastoconidia. Some of them may form pseudohyphae, which are chains of elongated yeast cells (Kurtzman et al. 2011). All yeast isolates were further confirmed and identified using molecular techniques. Yeast colonies were purified using the streak plate method and sub‐cultured on the SDA (HiMedia, India) agar and incubated for 48 h at 37°C. DNA extraction was performed from a single fresh colony. The yeast culture stocks were stored in 40% glycerol (Merck, Germany), with Sabouraud Dextrose Broth (SDB) (HiMedia, India) at −80°C until use.

### 2.3 Yeasts Identification by Molecular Characterisation

Genomic DNA was extracted using the NaOH‐boiling method as described by (Dilhari et al. 2017; Pintong et al. 2023). Yeast isolates were pre‐cultured on the SDA agar plate for 48 h at 37°C. A full loop of yeast colony was transferred and washed by resuspending with 300 μL of sterilised Milli Q (MQ) water. The mixture was vortexed for 10–15 s and then centrifuged at 10,000 rpm, 4°C for 1 min. The supernatant was removed. One hundred microliters of 20 mM NaOH were then added. The mixture was vortexed vigorously for 1 min. Cell suspension was boiled at 95°C for 45 min, followed by placing on ice for 5 min. The suspension was centrifuged at 12,000 rpm, 4°C for 10 min. The supernatant containing DNA was transferred to a new sterile tube and stored at −20°C until use.

After DNA extraction, genomic DNA was amplified using the fungal‐universal primer pair ITS5 (5′‐GGAAGTAAAAGTCGTAACAAGG‐3′) and ITS4 (5′‐TCCTCCGCTTATTGATATGC‐3′) (Internal Transcribed Spacer region) (Table S3) (Martin and Rygiewicz 2005; Schoch et al. 2012). The PCR mixture was prepared in 25 μL per reaction containing 2× Green PCR Master Mix (0.2 mM of each dNTP at 1× and 3 mM MgCl_2_ at 1×) (BR0100401; biotechrabbit GmbH, Germany), 10 μM ITS4 rDNA primers, 10 μM ITS5 rDNA primers, PCR‐grade water, and DNA template (10 ng/μl). PCR amplification was performed as follows: an initial denaturation step at 95°C for 5 min, followed by 35 cycles of amplification at 96°C for 30 s, annealing at 55°C for 30 s, and extension at 72°C for 30 s, with a final extension step at 72°C for 5 min. The ITS PCR amplicons were investigated by 1.5% (w/v) LE agarose gel electrophoresis. The gel was visualised, and images were taken using the G‐Box BioImaging System (Syngene Synoptics, UK) with GeneSnap software. The image was analysed using GeneTools software (Syngene, Synoptics, UK) to determine the size of the bands.

### 2.4 DNA Sequences of Environmental Yeast Isolates

The ITS PCR amplicons were purified using the FavorPrep GEL/PCR Purification Kit (FAVORGEN Biotech Corporation, Taiwan). The nucleotides of amplicons were sequenced (BioBasic Service, Singapore). Raw sequences were edited by BioEdit software (http://www.mbio.ncsu.edu/BioEdit/bioedit.html). For yeast identification, all contig sequences of ITS regions were compared with the reference sequences in GenBank using the BLASTn tool (https://blast.ncbi.nlm.nih.gov/Blast.cgi). According to a standard criterion, more than 95% sequence identity was used for yeast genus identification, whereas sequence identity of 97%–100% was used as a cut‐off for species identification.

### 2.5 Phylogenetic Analysis

To assess the genetic diversity of yeast isolations, the contig sequences were aligned using CLUSTALW in MEGA (Molecular Evolutionary Genetic Analysis) software, X version (www.megasoftware.net). The phylogenetic tree was constructed as unrooted. The phylogenetic tree was analyzed using the IQ‐TREE program (http://www.iqtree.org/). The tree was inferred using the maximum likelihood (ML) method. The best‐fit models for the data set were selected according to the Bayesian Information Criterion (BIC) of IQ‐TREE. The lowest BIC model was chosen to generate the construction of ML phylogenetic trees. The bootstrap resampling analysis involving 1000 replicates was applied to evaluate the topology of the trees and to estimate the phylogram stability. Then, the Newick format of tree files was uploaded to the Interactive Tree Of Life or iTOL program (https://itol.embl.de/) to visualize, annotate, and manage the phylogenetic tree. The tree was generated as a cladogram with branch lengths and bootstrap values ≥ 50% shown above the branches.

## 3 Results

### 3.1 Environmental Yeasts Isolation and Identification

**FIGURE 3:**
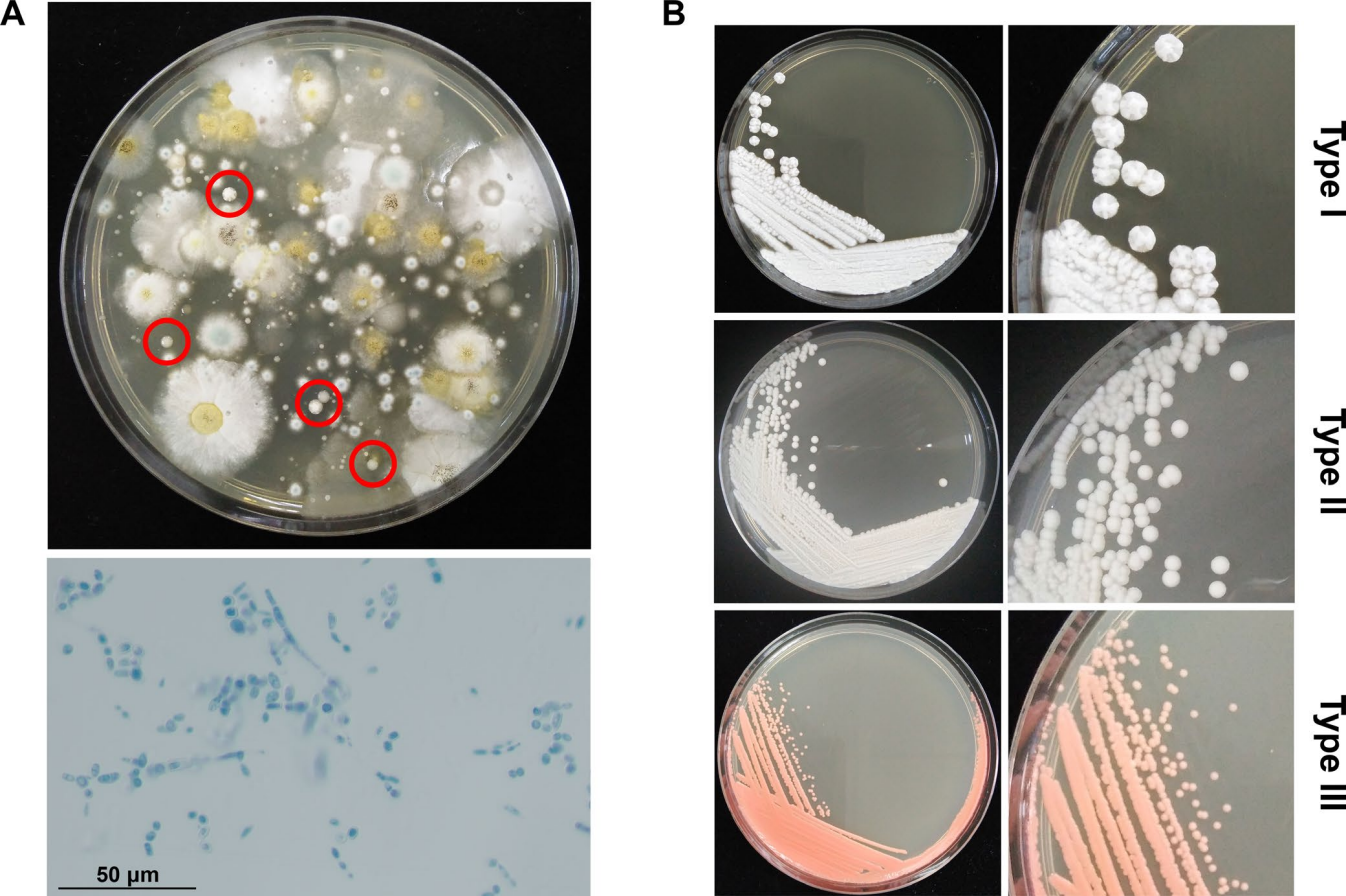
Isolation and identification of isolates from recreation areas. (A) The selected colonies of yeast‐like fungi (red circles) on SDA agar after incubation at 37°C for 5 days, and a representative example of yeast isolates with LPCB staining observed under microscopic (400X). (B) Three distinct colony morphologies of yeast isolates on SDA.

A total of 158 samples (120 samples from soil and 38 samples from water) were collected from the 12 public parks in Bangkok, Thailand. Yeast colonies were obtained from 84 of a total of 158 samples (53.16%) (Table 1). After examination for their characteristics by macroscopic and microscopic means (Figure 3A), the total of 321 yeast‐like isolates from 84 positive samples on SDA revealed three distinct morphologies (Figure 3B); (i) white to cream‐coloured, wrinkled, raised, and folded or cerebriform colonies; (ii) round, convex, smooth, creamy or white colonies; and (iii) round, smooth, mucoid, orange‐ or salmon‐coloured colonies.

**TABLE 1:** Number of sample collection and yeast abundance from each recreation area.

Place of sample collected	Park code	Numbers of collected samples			Number of samples containing yeasts			Percentage of samples containing yeasts	Number of yeast isolates using morphological identification	Number of yeast isolates using ITS region amplification^#^	Mean^a^	Std. dev.^a^
		Soil	Water	Total	Soil	Water	Total*					
Garden 60th Anniversary Queen Park	GA	10	5	15	10	4	14	93.33%	48	43	2.867	3.759
Phanphirom Park	PP	10	—	10	8	—	8	80%	24	10	1	2.494
Princess Mother Memorial Park	PM	10	2	12	7	2	9	75%	21	8	0.667	0.985
Thonburirom Park	TB	10	8	18	10	3	13	72.22%	56	12	0.667	1.283
Rama VIII Park	RM	10	3	13	6	3	9	69.23%	48	23	1.77	2.048
Chatuchak Park	CT	10	4	14	8	1	9	64.29%	64	45	3.214	7.073
Chaloem Prekiat 80 Phansa Park	CP	10	3	13	5	3	8	61.53%	14	14	1.077	1.188
Lumphini Park	LP	10	3	13	4	1	5	38.46%	11	11	0.846	1.676
Wachirabenchathat Park	WB	10	3	13	2	2	4	30.76%	14	14	1.077	2.753
Benchakitti Park	BK	10	1	11	2	—	2	18.18%	14	13	1.182	3.6
Suan Luang Rama IX Park	SL	10	3	13	1	1	2	15.38%	5	4	0.308	0.855
Santiphap Park	ST	10	3	13	—	1	1	7.69%	2	2	0.154	0.555
Total		120	38	158	63	21	84	53.16	321	199	—	—

^a^ Statistic data were calculated by one‐way ANOVA analysis using STATA. Mean and standard deviation were calculated based on the number of yeast isolations using ITS region amplification (#) and the total number of sampling sites (*) of each location to calculate the prevalence of yeast within that specific sampling area. The statistical analysis results from STATA were shown in Table S6.

The frequency of the yeast communities was different among the 12 public parks. The highest number of yeast‐like isolates using morphological identification were found in Chatuchak Park (64 isolates), Thonburirom Park (56 isolates), Garden 60th Anniversary Queen Park (48 isolates), and Rama VIII Park (48 isolates), whereas Santiphap Park showed the lowest number of yeast‐like isolates (2 isolates) (Table 1).

In this study, the ITS region was used as the target for yeast identification, as its variable size and sequence among different yeast species. The relatively high nucleotide substitution rate of this region enables the comparison of recently diverged taxa. Several previous studies also revealed that the ITS region is extremely variable, making it more suitable for identifying closely related yeast genera than other regions. In addition, the ITS region can be easily amplified by PCR and sequenced with ITS specific primers. Furthermore, we used the GenBank database to identify yeast genera in this study. In the GenBank database, the ITS region (the combination of ITS1 and ITS2) is preferred over other regions and has long been used as the universal marker for yeast identification. Due to its greater diversity and the large amount of information in the database, it has resulted in more categorised genera and more accurate identification (Abdel‐Sater et al. 2016; Badotti et al. 2017; Bellemain et al. 2010; Fajarningsih 2016). Thus, we decided to use the ITS region as a marker for yeast identification in this study.

A total of 321 yeast‐like isolates were confirmed by molecular diagnostics using ITS‐specific primers. The results showed that 199 out of 321 yeast‐like isolates were yeast positive and showed the amplicon size at approximately 400–900 bp. The results of ITS region amplification were quite similar to those of morphological identification. The highest number of positive results was found in Chatuchak Park and Garden 60th Anniversary Queen Park. The lowest number of yeast isolates was obtained from Santiphap Park, as shown in Table 1.

### 3.2 Environmental Yeast Abundance

To assess the abundance and diversity of 199 yeast isolates, the nucleotide sequences of positive PCR products were sequenced and compared with yeast sequences in NCBI database using Blast tool. Each genus was defined with cut‐offs including a percentage of identity with more than 95% and an E‐value equal to 0.00 (Table S4). According to the criteria, nucleotide sequence analysis of yeast isolates was classified into 22 genera as detailed in Table 2. The relative abundance of yeast isolates belonged to the phylum Basidiomycota (49.25%) and Ascomycota (45.23%) across all parks. The major genera were *Candida* (22.11%), followed by *Rhodotorula, Debaryomyces, Meyerozyma, Papiliotrema*, and *Trichosporon*. The analysis results also showed 5.53% of unclassified genera from total isolates and some less frequent genera detected such as *Moesziomyces, Kodamaea, Rhodosporidiobolus, Fereydounia, Ustilago, Pseudozyma, Kwoniella, Anthracocystis, Starmerella, Blastobotrys, Hannaella, Cystobasidium, Saitozyma, Lodderomyces, Pichia*, and *Wickerhamiella* (Table 2).

**TABLE 2:** Genera lists of yeasts and respective frequencies detected from environmental yeast isolated from recreation areas.

Genus	Number of isolates	Percent of isolates (relative abundance)	FO (%)^a^
Ascomycetous yeasts			
*Candida*	44	22.11%	52.38
*Debaryomyces*	19	9.55%	22.62
*Meyerozyma*	16	8.04%	19.05
*Kodamaea*	6	3.02%	7.14
*Starmerella*	1	0.50%	1.19
*Blastobotrys*	1	0.50%	1.19
*Lodderomyces*	1	0.50%	1.19
*Pichia*	1	0.50%	1.19
*Wickerhamiella*	1	0.50%	1.19
Basidiomycetous yeasts			
*Rhodotorula*	37	18.59%	44.05
*Papiliotrema*	15	7.54%	17.86
*Trichosporon*	11	5.53%	13.10
*Moesziomyces*	9	4.52%	10.71
*Rhodosporidiobolus*	6	3.02%	7.14
*Fereydounia*	4	2.01%	4.76
*Ustilago*	4	2.01%	4.76
*Pseudozyma*	4	2.01%	4.76
*Kwoniella*	3	1.51%	3.57
*Anthracocystis*	2	1.01%	2.38
*Hannaella*	1	0.50%	1.19
*Cystobasidium*	1	0.50%	1.19
*Saitozyma*	1	0.50%	1.19
Unclassified	11	5.53%	13.10
Total	199 isolates		
Genera richness	22 genera		

^a^ %FO; Frequency of occurrence (%) was calculated as the number of samples and a proportion of the number of samples in which yeasts occur.

### 3.3 Yeast Diversity Assessment

**FIGURE 4:**
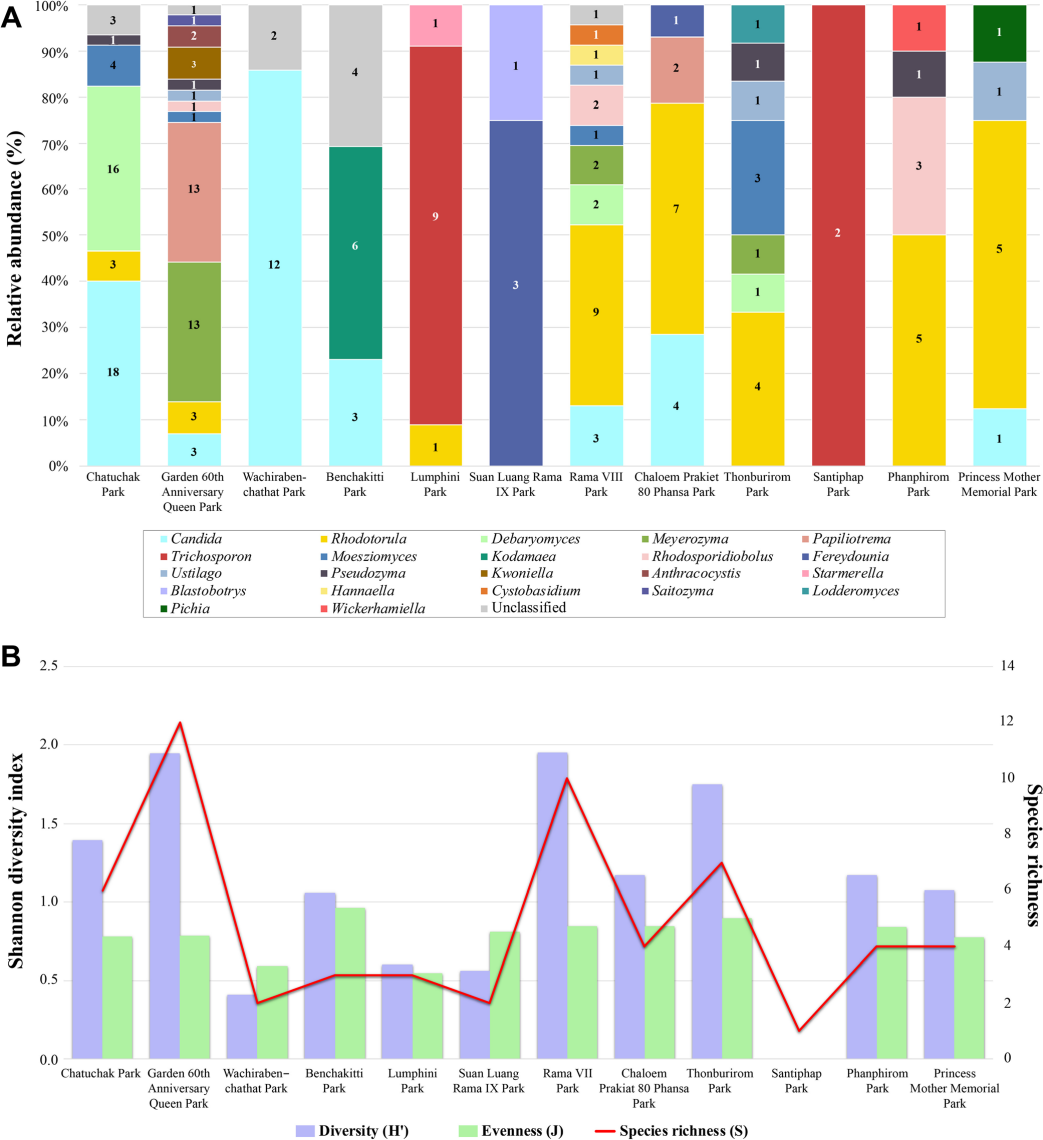
Frequency and distribution of environmental yeast species isolated from recreation areas in Bangkok. (A) Yeast community composition based on relative abundances of yeast genera in the 12 recreation areas. (B) Bar chart of species richness, Shannon diversity index, and evenness of yeast isolates in each park. BK, Benchakitti Park; CP, Chaloem Prekiat 80 Phansa Park; CT, Chatuchak Park; GA, Garden 60th Anniversary Queen Park; LP, Lumphini Park; PM, Princess Mother Memorial Park; PP, Phanphirom Park; RM, Rama VIII Park; SL, Suan Luang Rama IX Park; ST, Santiphap Park; TB, Thonburirom Park; WB, Wachirabenchathat Park.

Furthermore, the diversity of yeast genera showed different patterns in each collecting site. The distribution pattern of yeast was very diverse in the Garden 60th Anniversary Queen Park, Rama VIII Park, Thonburirom Park, and Chatuchak Park, indicating a greater species richness and relative species abundance of yeasts (Table 3 and Figure 4A). The diversity of yeast isolates in each public park also showed the differences according to the Shannon index, with the index of the yeast isolates ranging from 0.00 to 1.95. The Shannon index of yeast isolates in Garden 60th Anniversary Queen Park (12 genera) and Rama VIII Park (10 genera) was higher than that of yeast isolates from other parks (Table S5). Moreover, the species richness, diversity, and evenness of yeast isolated from the Garden 60th Anniversary Queen Park (GA) were highest. In contrast, those index values of yeast isolates in Santiphap Park (ST) showed as 0.00, indicating no diversity (Figure 4B).

**TABLE 3:** The correlation between yeast diversity indices of yeast genera and density of human use in different recreation areas.

Recreation areas	Area sizes (km^2^)	No. of visitors in 2019–2021	Average number of visitors per day (2019–2021)	Population density in recreation areas (people per km^2^)	Abundance (number of yeast isolates)	Yeast diversity		
						Species richness (*S*)	Shannon Diversity Index (*H*′)	Evenness (*J*)
Large Park (area size > 0.05 km^2^)								
Suan Luang Rama IX	0.80	6,658,399	6080.73	7600.91	4	2	0.562	0.811
Wachirabenchathat	0.60	6,374,051	5821.05	9701.75	14	2	0.410	0.592
Lumphini	0.58	7,863,335	7181.13	12,467.24	11	3	0.600	0.546
Benchakitti	0.51	2,253,360	2057.86	4019.26	13	3	1.058	0.963
Chatuchak	0.25	4,544,113	4149.87	16,733.37	45	6	1.395	0.779
Thonburirom	0.10	1,609,551	1469.91	14,699.10	12	7	1.748	0.898
Garden 60th Anniversary Queen	0.09	4,848,223	4427.60	52,089.42	43	12	1.948	0.784
Rama VIII	0.06	529,610	483.66	8636.82	23	10	1.952	0.848
Small Park (area size < 0.05 km^2^)								
Chaloem Prekiat 80 Phansa	0.03	1,222,481	1116.42	39,872.18	14	4	1.171	0.845
Santiphap	0.03	979,492	894.51	27,953.54	2	1	0.000	0.000
Phanphirom	0.02	ND	ND	—	10	4	1.168	0.843
Princess Mother Memorial	0.01	ND	ND	—	8	4	1.074	0.774

*Note:* Species richness = calculation for the number of observed species in each location. Shannon diversity index and evenness were used to estimate species diversity.

Abbreviation: ND, no data collection.

Garden 60th anniversary queen park, Chatuchak Park, and Thonburirom Park are large public parks (area size > 0.05 km^2^) and also have a high density of human activities at 52,089.42, 16,733.37, and 14,699.10 people/km^2^, respectively. On the contrary, the results also showed that some of the large public parks had low yeast proportions and diversity, such as Suan Luang Rama IX Park (0.80 km^2^), Wachirabenchathat Park (0.60 km^2^), Lumphini Park (0.58 km^2^), and Benchakitti Park (0.51 km^2^) (Table 3). These findings indicated that park size has no direct impact on the yeast abundance and diversity in recreation areas. In addition to park size, we found that some large parks with low yeast diversity also showed low human population density in their areas. These results suggest that the human population density of each park may affect the yeast abundance. We also investigated that Chaloem Prekiat 80 Phansa Park is a small area that showed a higher proportion of yeasts than that of some large public parks. This park is located in an area with a population density of 39,872 people/km^2^, which might result in a high yeast abundance and diversity.

Moreover, other factors might affect yeast diversity, including the number of human visits per day and the surrounding environment. As in Rama VIII Park (RM), while having a low density of human use, this park also presented a high occurrence and diversity of yeasts. From our location survey, this park is located near the Chao Phraya River, the main river of Bangkok (Figure S2G). This park also uses water from the Chao Phraya River as the main water supply for land and garden maintenance, which may be a factor causing a diversity of yeast in the area (Table 3). Besides, our investigations also revealed that the recreation areas with high ratios and diversity of yeast, including Garden 60th Anniversary Queen Park (GA), Chatuchak Park (CT), Thonburirom Park (TB), and Rama VIII Park (RM), are typically located within a 1 km radius of crowded and active areas (Figure S2). These parks are surrounded by high‐traffic areas with a large number of human uses, such as fresh markets, flea markets, pet markets, plant markets, street food markets, schools, and transportation hubs (Table 4). Therefore, our findings suggested that there are several factors, such as park landscape, park maintenance, environment, human behaviour, and surrounding area, that may affect yeast diversity and abundance.

**TABLE 4:** The correlation between diversity index of yeast and surrounding localities at each recreation areas.

Reacreation areas	Surrounding localities (Radius 1 km)	Yeast abundance	Yeast diversity		
			Species richness (*S*)	Shannon diversity index (*H*′)	Evenness (*J*)
Garden 60th Anniversary Queen	Flea market and fresh market	43	12	1.948	0.784
Chatuchak	Animal market, plant market, weekend market, street food market, transportation hub, and tourist spot	45	6	1.395	0.779
Rama VIII	Chao Phraya River, school, fresh market, and slum area	23	10	1.952	0.848
Thonburirom	University (King Mongkut's University of Technology Thonburi)	12	7	1.748	0.898
Chaloem Prekiat 80 Phansa	Office building and government area	14	4	1.171	0.845
Wachirabenchathat	Toll road	14	2	0.41	0.592
Benchakitti	Office building	13	3	1.058	0.963
Lumphini	Office building and mall	11	3	0.6	0.546
Phanphirom	Toll road	10	4	1.168	0.843
Princess Mother Memorial	Village	8	4	1.074	0.774
Suan Luang Rama IX	Village	4	2	0.562	0.811
Santiphap	Office building and mall	2	1	0	0

**FIGURE 5:**
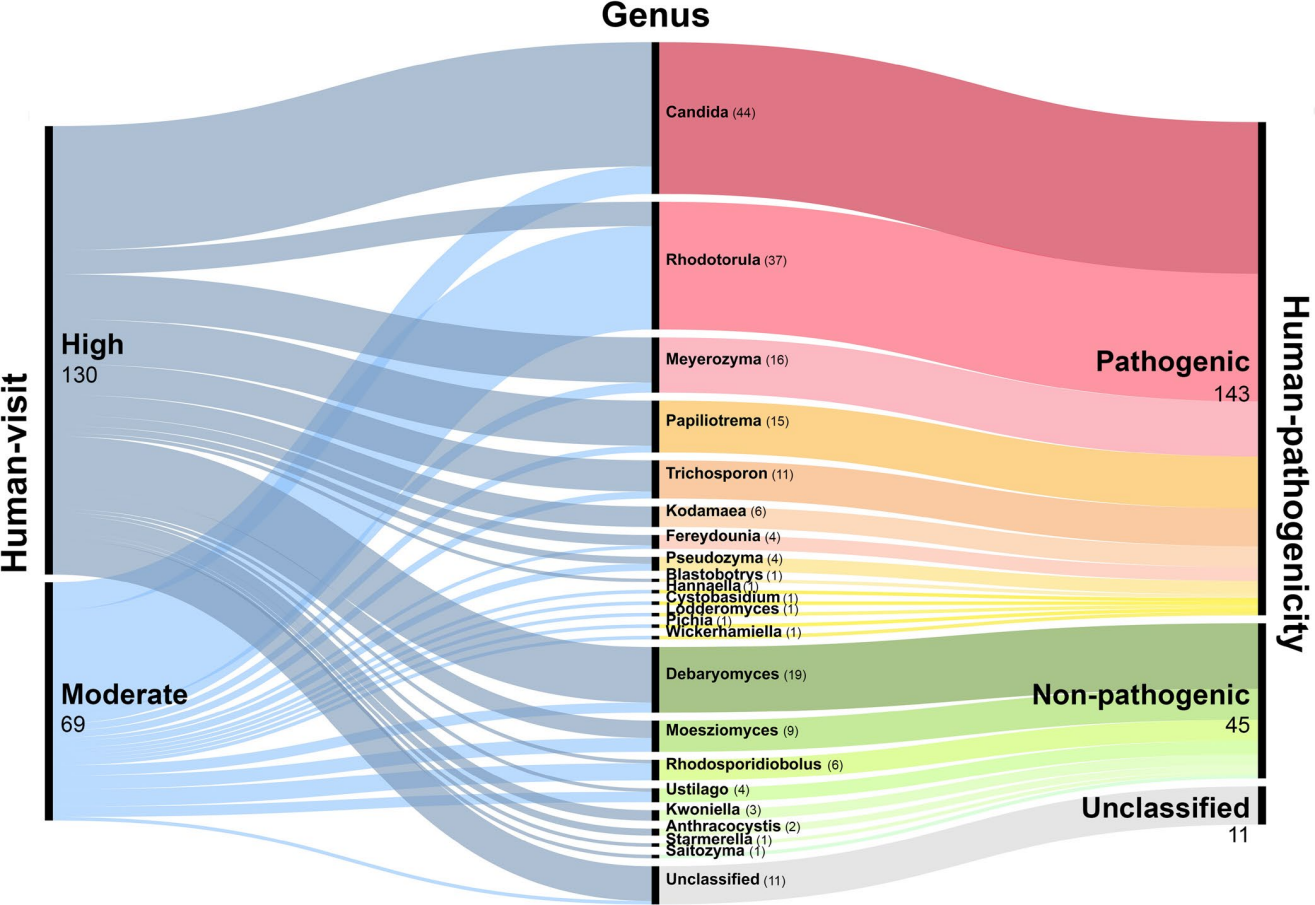
Sankey diagram represents the relationship of yeast isolates in human pathogenic with human‐visit of recreation areas. The width of each connection is proportional to the number of yeast isolations. The flow in left represents level of human‐visits in each recreation areas (blue colour). The bold black line in the centre represents the number of yeast genera that identified based on ITS region sequencing analysis. The flow in right represents the yeast pathogenicity. Red to yellow flows represent as human pathogenic, whereas, green flows represent as non‐pathogenic in human. In this figure, we classify yeast genera as “human pathogenic” if it has been described to cause diseases in humans, whether as a true or an opportunistic pathogen. The term “non‐human pathogenic” refers to yeast genera that have never been recorded to cause human diseases. The levels of human visit were annually summarised and divided into two groups as high human‐visited (> 400,000 people) and moderate human‐visited (100,000–400,000 people) areas (data from Department of Environment, Bangkok, Thailand; updated on December, 2023). The Sankey diagram was generated using RAWGraphs 2.0 (https://app.rawgraphs.io/).

From all 12 public parks, a total of 199 environmental yeasts, 143 isolates were identified as the human pathogenic yeasts and 45 isolates were classified as non‐pathogenic yeasts. We classified human pathogenic and non‐pathogenic yeasts according to their pathogenicity evidence for each yeast genus. We define yeast genera as “human‐pathogenicity” if they are discovered to have the capability to cause disease in people, whether true or opportunistic pathogens. The “non‐human pathogenicity” group is referred to yeast genera that have not previously been noted to cause diseases in humans. In Table 5, we found the higher ratio of pathogenic yeast isolates than nonpathogenic yeasts in all study areas. To evaluate the association between human activities and yeast community, our study areas were divided into two groups as high human‐visited (> 400,000 people annually) and moderate human‐visited (100,000–400,000 people annually) areas. The results showed that high human‐visited parks had 90 isolates of pathogenic yeasts, which is more than that in moderate human‐visited parks (53 isolates) (Table 5). However, the results of this study cannot indicate that the number of pathogenic yeasts is related to the level of human visits in each park. Moreover, the correlation of the prevalence of pathogenic yeasts and human visits in each park using Sankey analysis was performed (Figure 5). This diagram illustrates the connection of human visits, the occurrence and diversity of environmental yeasts, and the human pathogenicity of yeasts. The flow in blue colour represents the level of human visitors. At the left side, the red to yellow flow represents pathogenic yeasts, whereas the green flow represents non‐pathogenic yeasts in humans. The results showed a difference in patterns and trends of yeast between high and moderate human‐visited parks. The findings also demonstrated the large proportion of pathogenic yeasts in high human‐visited parks. These results revealed the correlations between pathogenic yeasts and human‐visited levels, which could pose a concern to humans who commonly use recreation areas. However, there are several factors that may affect pathogenic yeast patterns.

**TABLE 5:** The correlation between the number of pathogenic yeast isolates and the level of human visits annually in different recreation areas.

Recreation areas	Number of yeast isolates			
	Pathogenic yeasts	Non‐pathogenic yeasts	Unclassified	Total yeast isolates
High human‐visited (> 400,000 people)	90	30	10	130
Garden 60th Anniversary Queen	33	9	1	43
Chatuchak	22	20	3	45
Wachirabenchathat	12	—	2	14
Lumphini	10	1	—	11
Benchakitti	9	—	4	13
Suan Luang Rama IX	4	—	—	4
Moderate human‐visited (< 400,000 people)	53	15	1	69
Rama VIII	16	6	1	23
Chaloem Prekiat 80 Phansa	14	—	—	14
Thonburirom	7	5	—	12
Phanphirom	7	3	—	10
Princess Mother Memorial	7	1	—	8
Santiphap	2	—	—	2

### 3.4 Phylogenetic Analysis of Yeast Isolates

**FIGURE 6:**
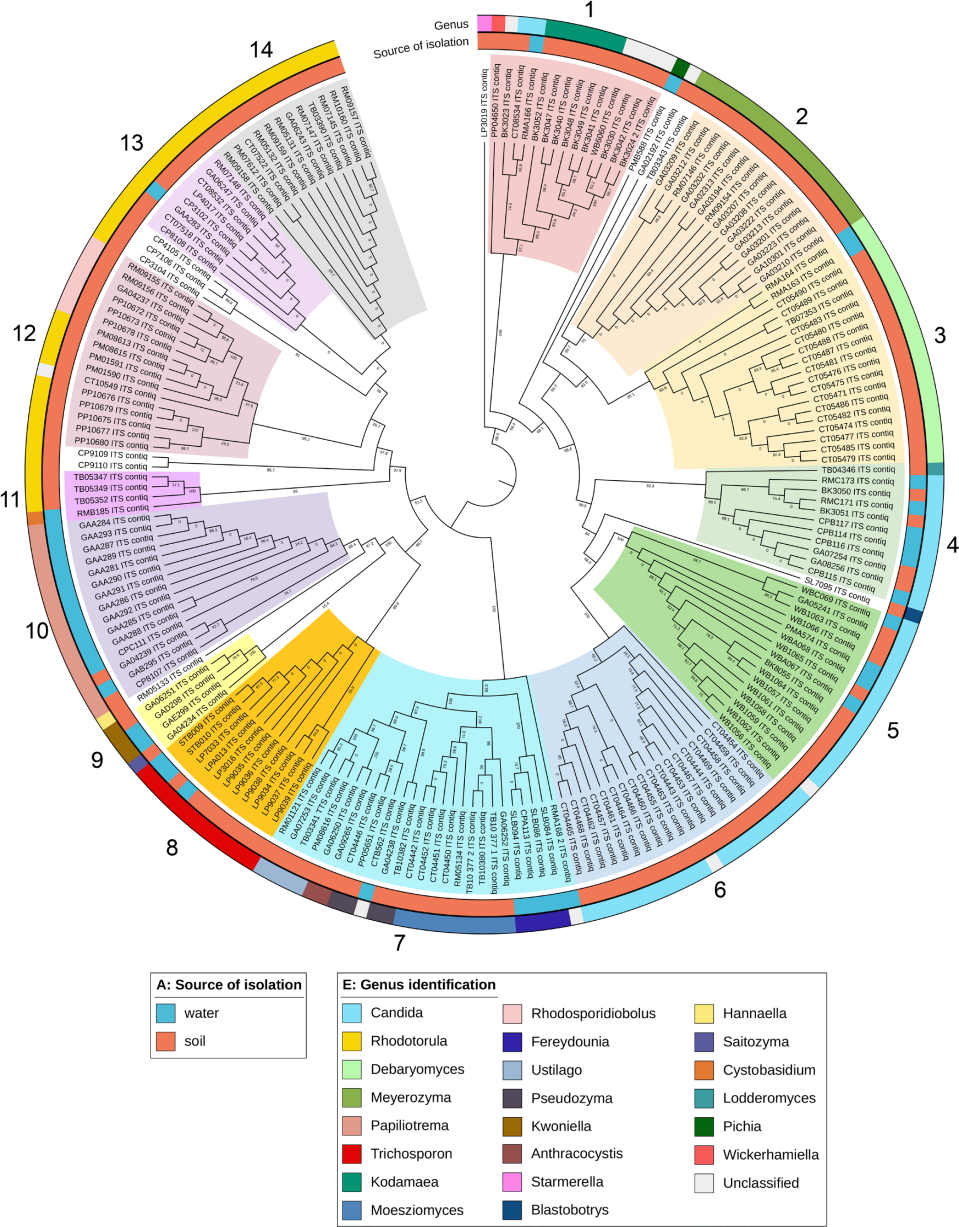
The maximum likelihood (ML) phylogenetic tree of diversity of yeast isolates from recreation areas. ML tree was constructed from 1000 bootstraps with TPM3 + F + R4 models based on BIC scores (45297.5097). Log‐likelihood of the tree is −21190.7374. Different genera were indicated with clade colours. Five outer rings showed metadata of each isolate and were labelled as specific colours, including source of isolation (A), Division (B), Class (C), Family (D), and Genus (E).

The nucleotide sequence from 199 yeast isolates was also used to evaluate the yeast diversity. All contig sequences from the ITS region were constructed into a maximum likelihood phylogenetic tree using IQ tree software and were visualised with metadata using the iTOL program. The bootstrap value with 1000 replications was performed. As a result, the phylogenetic tree was constructed, and yeast isolates were separated into 14 clades with high bootstrap support (> 50%) (Figure 6). The phylogenetic tree of environmental yeast isolates revealed that the isolates were clustered in grouping clades of the same and closely resembling genera. For example, all isolates of *Meyerozyma* (Clade 2), *Debaryomyces* (Clade 3), *Trichosporon* (clade 8), and *Papiliotrema* species (Clade 10) were clustered in their clade and completely separated from other genera. In addition, some genera were separated into several clades, such as *Candida* and *Rhodotorula* species. *Candida* isolates, in this study, belonged to Clades 4–6, while *Rhodotorula* isolates were clustered in Clades 11–14. These results revealed that the genera of *Candida* and *Rhodotorula* typically contain various type strains and are highly variable, resulting in diversification into multiple clades. However, in some clades, the different genera shared a common branch, indicating closely related genera. For example, Clade 7 and Clade 1 showed several genera clustered together with 1000 bootstrap analyses, indicating that these genera were related species and had closely related relationships based on the ITS region. Furthermore, the ITS region tree also revealed that some related genera, such as *Rhodotorula* and *Rhodosporidiobolus* in Clade 12, were clustered together, indicating the evolutionary history and relationships between these two genera (Figure 6).

**FIGURE 7:**
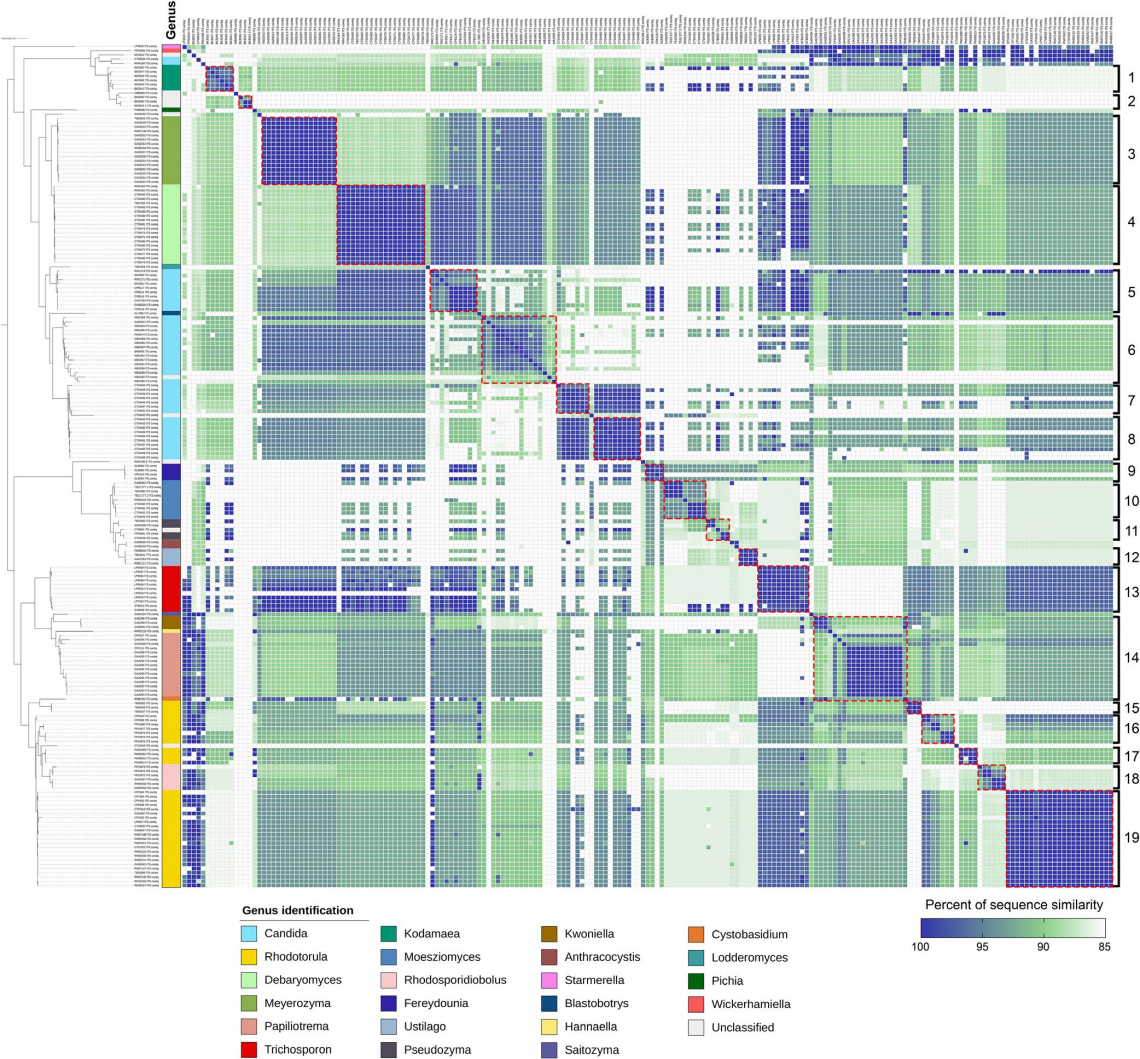
The maximum likelihood (ML) phylogenetic tree and pairwise sequence similarity matrix of environmental yeast isolates based on ITS region. Yeast isolates were separated into 19 groups and 15 singletons with the percentage of similarity at 90%–100% as a cut‐off. Groups of isolates were labelled with red‐coloured boxes.

To evaluate the percentage of similarity among 199 nucleotide sequences of environmental yeast isolates, the identity matrix was generated using phylogenetic tree ordering (Figure 7). The matrix result showed the relationships and similarities among our yeast isolates, with sequence similarity ranging from 85% to 100% based on BLASTN all‐versus‐all. Based on the percentage of similarity at 90%–100%, yeast isolates were divided into 19 groups and 15 singletons. *Meyerozyma* (Group 3), *Debaryomyces* (Group 4), and *Trichosporon* (Group 13) were entirely separated from the others, with 90%–100% similarity within their groups. In group 14, *Kwoniella* and *Papiliotrema* species were clustered together, whereas *Rhodotorula* and *Rhodosporidiobolus* species were clustered in group 18. The results from groups 14 and 18 indicated that yeast genera in these two groups were defined as closely related genera based on the ITS region. Besides, some genera, such as *Candida* species (Groups 5–8) and *Rhodotorula* species (Groups 15–19), have been separated into multiple groups, showing that their species are quite varied and highly diverse. From our findings, the matrix analysis based on the ITS region revealed that the majority of our isolates had more than 85% similarity. The closely related genera were shared with ≥ 90% similarity within their groups.

Although using nucleotide sequences of the ITS region has been possible to recognize the genus of yeasts. On the other hand, our study also found that some genera did not belong to the same groups and were distributed to multiple groups. Some isolates were separated as singletons or did not share any similarities within the same genus. Moreover, some isolates in our study were unsuccessfully classified, resulting in unclassified or uncultured fungus (Table S4). These findings suggested that the ITS regions can be used to identify yeast genera. However, the ITS region could not be completely identified and classified for all yeast genera, especially in highly variable strains. Hence, several alternative regions might be applied as secondary regions for identification of the vast majority of Ascomycota and Basidiomycota genera. Therefore, the study of the novel alternative regions in further studies could be useful and effective for finer‐scale species‐level identification of environmental yeasts.

## 4 Discussion

Many countries have recently shown an increased interest in environmental yeast diversity. More than 60% of the environmental yeast diversity reports come from tropical and subtropical regions (Boekhout et al. 2021; Rosa et al. 2023). Several articles have demonstrated that various yeast species have been found in soil and water of public urban locations such as children's playgrounds, parks, and beaches (Wojcik et al. 2013; Moazeni et al. 2022; Biedunkiewicz and Góralska 2016; Brandão et al. 2021; Chen et al. 2009; Maciel et al. 2019; Shah et al. 2011; Yurkov et al. 2012). In Thailand, yeast diversity has been assessed in a variety of natural resources, including mangroves, forests, farms, oceans, waterfalls, caves, and pigeon excreta (Into et al. 2020; Kaewkrajay et al. 2020; Kaewwichian and Khamthaiklang 2022; Kanpiengjai et al. 2023; Nualmalang et al. 2023; Rosa et al. 2023; Sapsirisuk et al. 2022; Satianpakiranakorn et al. 2020). A study by Sapsirisuk et al. found that yeasts from a forest in northern Thailand had species of *Lipomyces*, *Trichosporon, Candida, Pichia*, and *Dipodascus* species (Sapsirisuk et al. 2022). Whereas the publication by Kaewwichian and Khamthaiklang reported that *Candida, Galactomyces, Pichia*, and *Trichosporon* were the most common yeast species found in mangrove environments (Kaewwichian and Khamthaiklang 2022). Kaewkrajay and their colleague investigated 34 yeast strains in the western part of the South China Sea in the Gulf of Thailand and revealed a significant prevalence of *P. laurentii* (Kaewkrajay et al. 2020). However, no yeast diversity surveys are conducted in public parks.

Normally, the ITS region and D1/D2 are both commonly used for yeast identification. In general, the ITS region is the spacer that is located between SSU and LSU, whereas the D1/D2 region is located in LSU. ITS regions are generally more variable than the D1/D2 region, meaning more sequence divergence among species. Previous studies revealed that the ITS region is hyper‐variation, which is suitable for classification of closely related fungal genera. While the D1/D2 region has less variability and diversity than ITS (Fajarningsih 2016; Op De Beeck et al. 2014; Raja et al. 2017; Zaher and Mahmoud 2015; Aydin et al. 2019). As in a previous study, the sequence variation within the D1/D2 and ITS regions between two closely related species of the Yamadazyma genus was compared. The results revealed that the ITS region is more variable than that of the D1/D2 domain (Groenewald et al. 2011). Moreover, in our study, we used the nucleotide sequences from the GenBank database for identification of yeast genera. In the GenBank database, the ITS region is more popular than the D1/D2 region due to its higher level of variation, allowing for more exact discrimination, especially for very closely related genera. Although the D1/D2 can be used for broader identification, the ITS region offers a more sensitive approach for yeast identification with more accuracy (https://www.ncbi.nlm.nih.gov/bioproject/PRJNA177353).

According to previous studies, yeasts in urban recreation areas can survive on environmental materials and spread easily to animals or humans (Akinbobola et al. 2023). Thus, the focus of this study was initially on the diversity of yeast in urban recreation areas or public parks in Bangkok, Thailand. From previous studies, they suggested that the diversity of yeast can vary due to several factors such as country, environment, location, humidity levels, temperature, and human activities (Akinbobola et al. 2023; Brandão et al. 2021; Frac et al. 2018). According to some studies, the population of environmental yeasts in the soil and water is lower than that of bacteria and fungi (El‐Tarabily and Sivasithamparam 2006). The diversity and abundance of yeasts can differ depending on the soil and water types. For example, several studies discovered that forest soils have distinct yeast community patterns compared to grassland soils (Devi et al. 2025). A previous study has also indicated that the soil yeast population correlates with the organic content in soil, resulting in a greater yeast population in rhizosphere soil than in bulk soil (van den Heever et al. 2025). The study of Slavikova and Vadkertiova concluded that the average yeast population in agricultural soils was lower than in forest soils, as a result of agricultural preparation of soil, such as digging, stirring, and overturning (Slavikova and Vadkertiova 2003). They also proposed that semi‐arid soils in hot regions may have lower yeast abundance due to low nutrient concentrations, low water content, high temperatures, and limited plant cover (Slavikova and Vadkertiova 2003). In addition, the existence of yeasts in water has few studies. Most of them focus on polluted water. They noted that water pollution, the percentage of human use, and the surrounding environment of water sources are factors that can impact the occurrence of yeasts in water (Nagahama 2006; Libkind et al. 2003). The abundance of yeasts in tropical rivers and lakes has been associated with freshwater contamination due to their location near forests, urban areas, and human living areas (Brandão et al. 2021). Furthermore, some yeast species isolated from freshwater environments have been implicated as opportunistic pathogens (Brandão et al. 2021; Monapathi et al. 2020; Monapathi et al. 2017).

In this study, our results also found differences in yeast community among study areas and collecting sites. Some public parks, such as Garden 60th Anniversary Queen Park and Chatuchak Park, have huge green areas with a lot of tree canopy and large water areas that may impact the occurrence and variety of yeasts. Meanwhile, Santiphap Park, which has the least proportion and diversity of yeasts, has relatively few green areas, and most of the area is made of concrete for jogging and exercising. Our findings also revealed that the water source in Santiphap Park is quite small. These factors might result in lower abundance and diversity of yeasts than in other parks. Therefore, these results and investigations suggested that the soil types, water sources, and surrounding environment in each park can also affect the yeast community (Figure S1).

On top of that, some studies showed that the ratio of yeast species is related to human activity in that area (Ayanbimpe et al. 2013; Wojcik et al. 2013; Dumontet et al. 2001; Slavikova and Vadkertiova 1997; Yurkov et al. 2012). In 2020, Satianpakiranakorn et al. discovered 35 yeast strains at Kuan Kreng and Rayong Botanical Gardens. They also concluded that yeasts were highly divergent in areas with intense human activities (Satianpakiranakorn et al. 2020). According to the results of the molecular identification, the various proportions of yeast isolates were associated with several factors, including the park sizes, human activities of each recreation area, human population density, as well as the surrounding environments. Similar results were found in this study that the highest species richness and diversity index of yeasts were identified in large public parks (Garden 60th Anniversary Queen Park and Chatuchak Park), which are intensely accessed by humans. The recreation areas with a high percentage of yeast isolates were large and had high human activities. On the other hand, the locations with the lowest percentage of yeast isolates were small, with few people visits and few activities. There are some large recreation areas in this study that had low yeast diversity due to their having a low amount of human visits. In addition, our findings also indicated that recreation areas with the highest concentration of yeast diversity are usually located in high‐traffic areas such as fresh markets, human communities, public transport hubs, city squares, and grand avenues. For example, Rama VIII Park (RM) located near a local market, residential area, as well as Chao Phraya River, showed the high abundance and diversity of yeasts. These factors may encourage a diversity of yeast communities in the park. Thus, we suggested that there are several factors that might be linked to the ratio of yeast abundance and diversity, such as the size of parks, park maintenance, location, surrounding environment, human visits, and human activities.

Our investigation also proved that yeast strains from recreations in urban areas were diverged more than those from areas with limited human activity, such as forests, mangroves, and oceans. Furthermore, several previous studies in Thailand also revealed that yeast strains in environments with high human activity are typically opportunistic yeasts (Into et al. 2020; Kaewkrajay et al. 2020; Kaewwichian and Khamthaiklang 2022; Kanpiengjai et al. 2023; Luplertlop et al. 2019; Paserakung et al. 2015; Satianpakiranakorn et al. 2020; Pintong et al. 2023). During our examination of urban public park soil and water in Bangkok, we observed 22 yeast genera, with *Candida* being the most abundant. This study's findings are consistent with those from prior global surveys in 2021. Samarasinghe et al. conducted global surveys and identified 90 kinds of environmental yeasts, with *Candida* being the most prevalent (Samarasinghe et al. 2021). Although *Candida* species represent the majority of yeasts from environmental materials in this study, other yeasts also found in public parks include *Rhodotorula, Debaryomyces, Meyerozyma, Papiliotrema*, and *Trichosporon* species, which have been reported as human pathogens. From the results, we investigated the differences in the percentage of pathogenic and non‐pathogenic yeasts among the 12 public parks of Bangkok. Our findings revealed that highly pathogenic yeast communities tend to be associated with human behaviours, indicating the relationship between pathogenic yeasts and human activities. From our investigation, we suggested that people who frequently use recreation areas are at high risk of yeast infection. Moreover, we also realized that soil and water in recreation areas might be another contact site between these pathogenic yeasts and humans.

Although yeasts can be classified by their genus and species using conventional techniques, yeast taxonomy has been continuously updated, and many novel genera have been recognised. As a result, conventional approaches are no longer sufficient to distinguish yeasts. Recently, DNA region detection has become a common tool for yeast identification. The variable regions, ITS region, are recently representative of consistent markers (Abdel‐Sater et al. 2016; Arabatzis et al. 2014; Colombo et al. 2011; Francisco et al. 2021; Guo et al. 2011; Sugita et al. 1998). Previous studies found that the medically significant species could be accurately identified using their ITS sequences (Leaw et al. 2006; Badotti et al. 2017; Fajarningsih 2016). Contrary to our investigation, the ITS region tends to be suitable for only yeast differentiation at the genus level. In this study, the ITS region of yeast isolates was successfully amplified in environmental yeast. However, there were some significant limitations when applied to yeast isolates in our study. Our results indicated that highly variable yeasts such as *Candida* were not completely clustered as a group and were distributed across several clades. Some related genera, such as *Kwoniella* and *Papiliotrema*, were unsuccessful in being separated and clustered in the same clade due to the significant similarity across closely related genera. Moreover, some of our isolates still showed as unclassified when compared to nucleotide databases. Consequently, we suggested that the ITS region was sufficient to distinguish environmental yeasts at the genus level. Moreover, our findings further suggested that the combination of several techniques may be useful for yeast identification. The use of different primer sets, alternative DNA regions, and novel DNA sequencing approaches might be interesting for the identification of environmental yeasts, especially high phylogenetic relation strains.

Finally, our study provided a conclusion about the relationship between environmental yeasts and human activities. Furthermore, this study might point to a correlation between human activities and pathogenic yeasts, including the association of these yeasts with human behaviour in that area.

## 5 Conclusion

We explore yeast abundance and diversity using soil and water samples obtained from 12 recreation areas in Bangkok, Thailand. Among 22 yeast genera in our study, the most frequently found genus was *Candida*. Our investigation also exhibited results in the identification of environmental yeasts using the ITS region. The results showed that the ITS region can be used to differentiate environmental yeasts at the genus level. However, some genera could not be classified using this region. From our findings, we found that park size and human activity may affect yeast abundance and yeast diversity. However, we discovered that several additional factors, such as the surrounding environment with high‐traffic areas, park landscapes, and water supplies for park maintenance, may also have an impact on the yeast community.

## Author Contributions

**Pantira Singkum:** writing – original draft, funding acquisition, writing – review and editing, formal analysis, validation, data curation, conceptualization, methodology, software, visualization. **Thanwa Wongsuk:** conceptualization, investigation, methodology, resources. **Potjaman Pumeesat:** conceptualization, investigation, methodology, resources. **Rattiya Cheewapat:** investigation, methodology. **Ingo Ebersberger:** investigation, formal analysis, software, visualization. **Rapee Thummeepak:** investigation, data curation, formal analysis, validation, software, visualization. **Amornrat Aroonnual:** conceptualization, investigation, writing – review and editing, data curation, funding acquisition, project administration, supervision, resources.

## Conflicts of Interest

The authors declare no conflicts of interest.

## Supporting information


**Figure S1:** Geographical distribution of the sampling points in each recreation areas; A = Santiphap Park (ST), B = Lumphini Park (LP), C=Benchakitti Park (BK), D=Wachirabenchathat Park (WB), E = Suan Luang Rama IX Park (SL), F=Chaloem Prekiat 80 Phansa Park (CP), G = Rama VIII Park (RM), H = Chatuchak Park (CT), I=Phanphirom Park (PP), J = Princess Mother Memorial Park (PM), K = Garden 60th Anniversary Queen Park (GA), and L = Thonburirom Park (TB). Blue pins corresponding to water‐sampling sites and yellow pins to soil‐sampling sites (Created by Google earth; https://earth.google.com/web/).

**Figure S2:** The mapping and surrounding localities around each recreation areas; A = Santiphap Park (ST), B = Lumphini Park (LP), C=Benchakitti Park (BK), D=Wachirabenchathat Park (WB), E = Suan Luang Rama IX Park (SL), F=Chaloem Prekiat 80 Phansa Park (CP), G = Rama VIII Park (RM), H = Chatuchak Park (CT), I=Phanphirom Park (PP), J = Princess Mother Memorial Park (PM), K = Garden 60th Anniversary Queen Park (GA), and L = Thonburirom Park (TB). This map created by Google earth; https://earth.google.com/web/.

**Table S1:** The temperature and humidity of each month in Bangkok, Thailand between 2019 and 2021 (station code: 455201). The information of temperature and relative humidity derived from Thai Meteorological Department.

**Table S2:** The global positioning system (GPS) coordination of sites determined using a Handy GPS application (free trial version, Binary Earth).

**Table S3:** Primers used to analyse the yeast isolations.

**Table S4:** Blast search results of yeast isolations in this study based on ITS region.

**Table S5:** The species richness and diversity indices of yeast genera in different recreation areas. Specie richness = calculation for the number of observed species in each location. Shannon Diversity Index and Evenness were used to estimate the species diversity.

**Table S6:** The statistical results of one‐way ANOVA using STATA analysis. The park codes were assigned as; 1 = Santiphap Park (ST), 2 = Lumphini Park (LP), 3 = Benchakitti Park (BK), 4 = Wachirabenchathat Park (WB), 5 = Suan Luang Rama IX Park (SL), 6 = Chaloem Prekiat 80 Phansa Park (CP), 7 = Rama VIII Park (RM), 8 = Garden 60th Anniversary Queen Park (GA), 9 = Thonburirom Park (TB), 10 = Chatuchak Park (CT), 11 = Princess Mother Memorial Park (PM), and 12 = Phanphirom Park (PP).

## Data Availability

The data that supports the findings of this study are available in the Supporting Information of this article.
