# Supplementary material for: Environmental Yeast Abundance and Diversity Assessment in Recreation Areas of Bangkok, Thailand
Source: Environ Microbiol Rep. 2025 Oct 21;17(5):e70212. doi: 10.1111/1758-2229.70212 (PMC12539370; doi:10.1111/1758-2229.70212)
Supplement: Supplementary file 2 — Figure S2: The mapping and surrounding localities around each recreation areas; A = Santiphap Park (ST), B = Lumphini Park (LP), C=Benchakitti Park (BK), D=Wachirabenchathat Park (WB), E = Suan Luang Rama IX Park (SL), F=Chaloem Prekiat 80 Phansa Park (CP), G = Rama VIII Park (RM), H = Chatuchak Park (CT), I=Phanphirom Park (PP), J = Princess Mother Memorial Park (PM), K = Garden 60th Anniversary Queen Park (GA), and L = Thonburirom Park (TB). This map created by Google earth; https://earth.google.com/web/. [file EMI4-17-e70212-s007.docx]

**Figure S2** The mapping and surrounding localities around each recreation areas; A=Santiphap Park (ST), B=Lumphini Park (LP), C=Benchakitti Park (BK), D=Wachirabenchathat Park (WB), E=Suan Luang Rama IX Park (SL), F=Chaloem Prekiat 80 Phansa Park (CP), G= Rama VIII Park (RM), H= Chatuchak Park (CT), I=Phanphirom Park (PP), J=Princess Mother Memorial Park (PM), K= Garden 60th Anniversary Queen Park (GA), and L= Thonburirom Park (TB). This map created by Google earth; <https://earth.google.com/web/>.

| (A) | 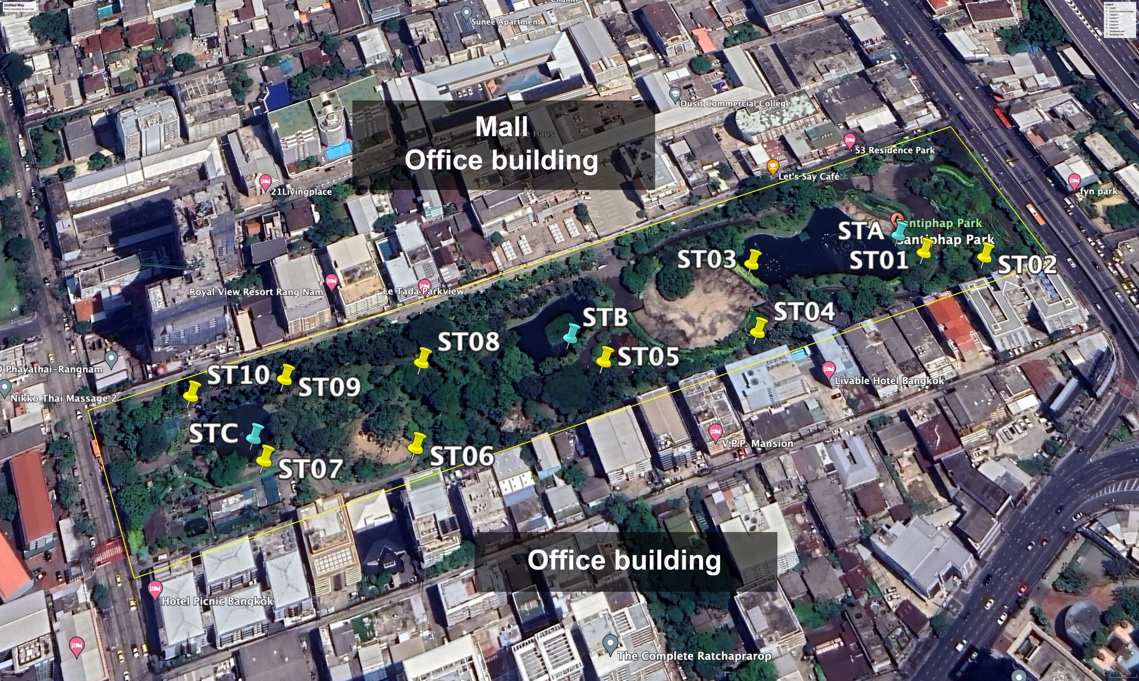 |
| --- | --- |
| (B) | 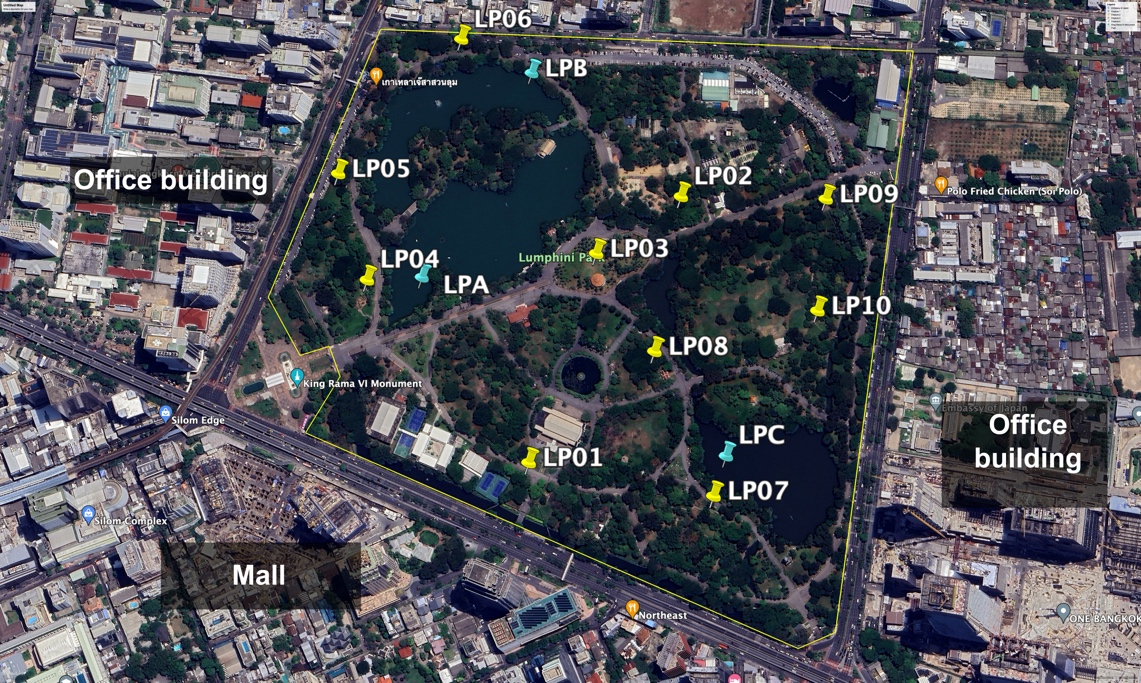 |
| (C) | 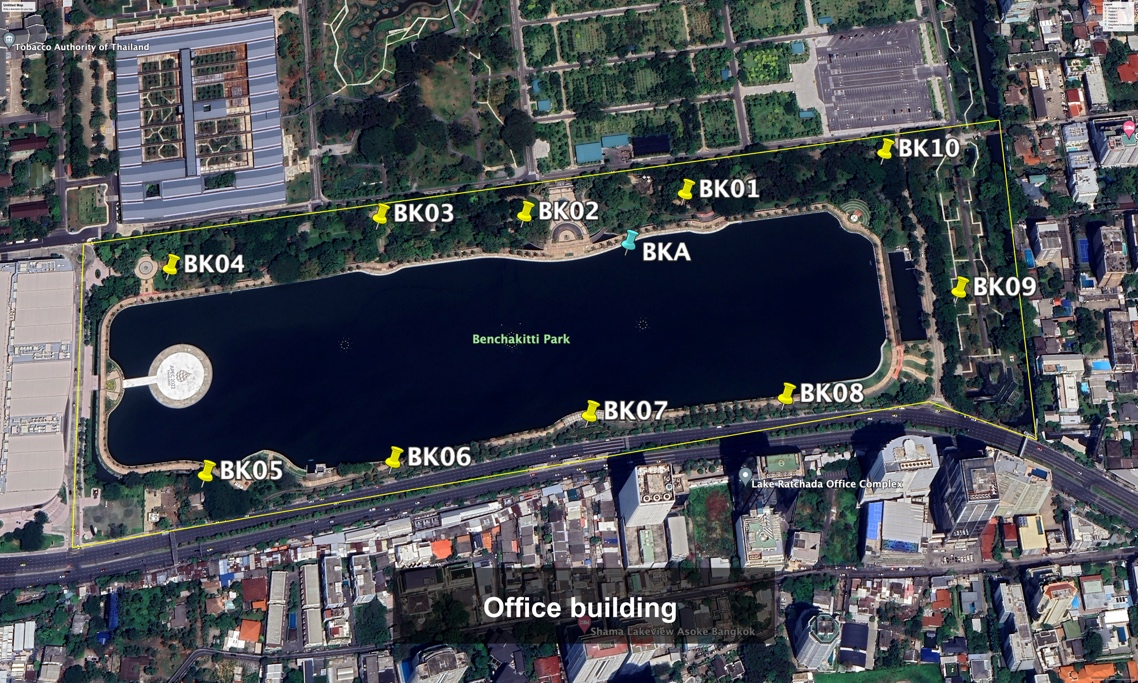 |
| (D) | 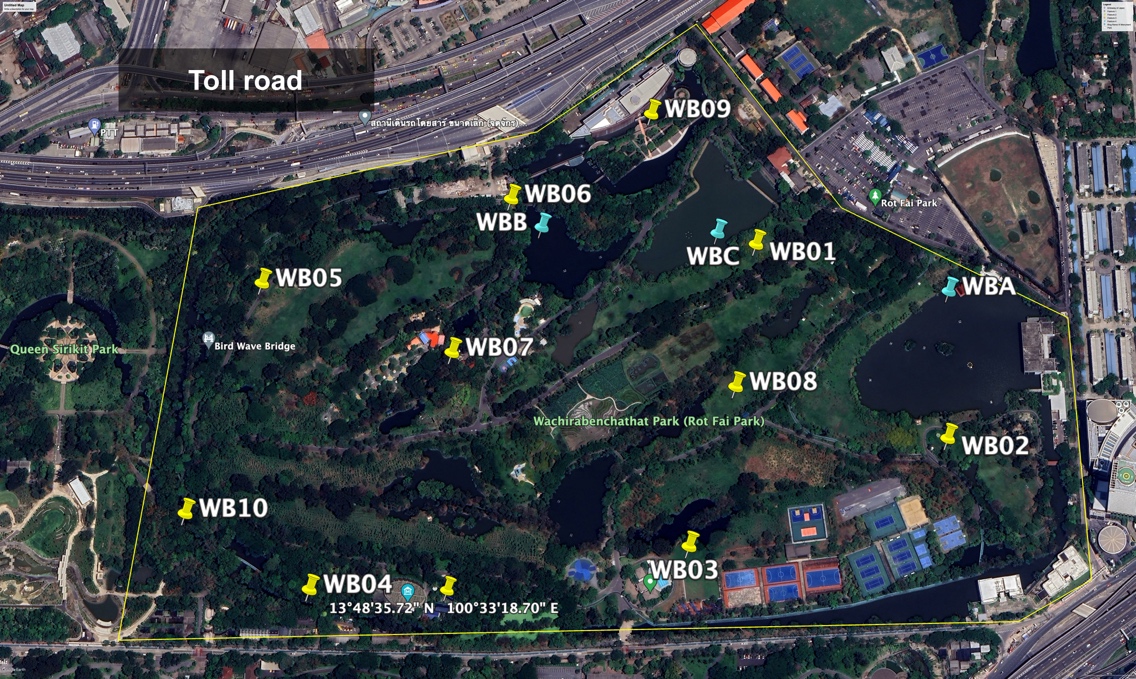 |
| (E) | 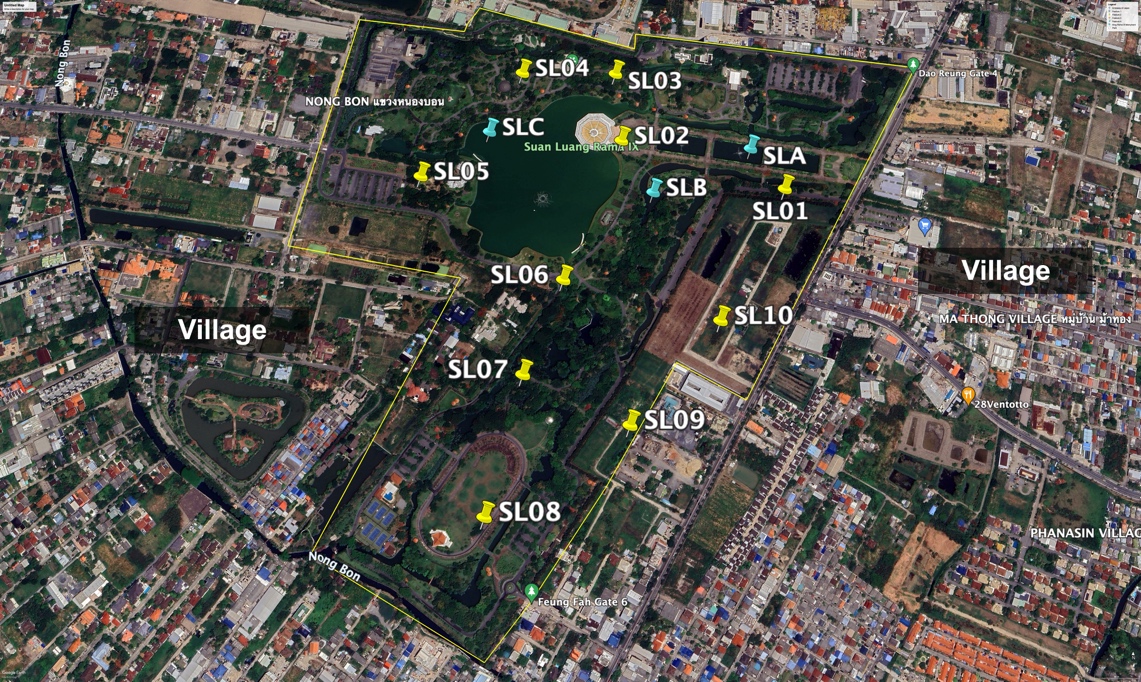 |
| (F) | 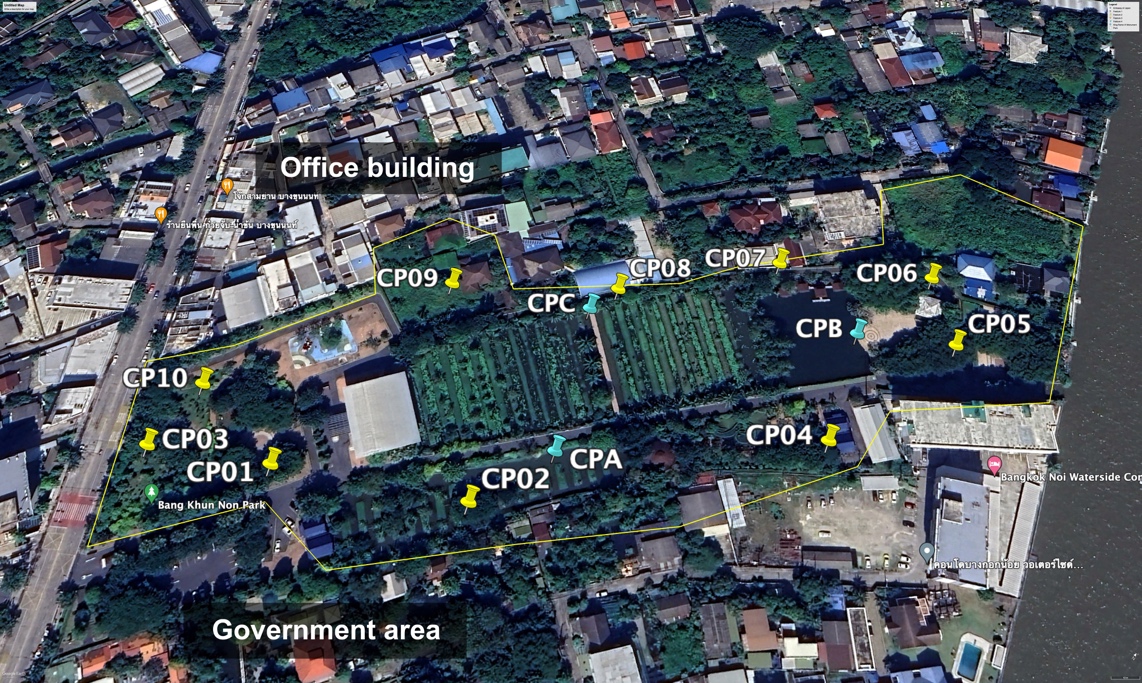 |
| (G) | 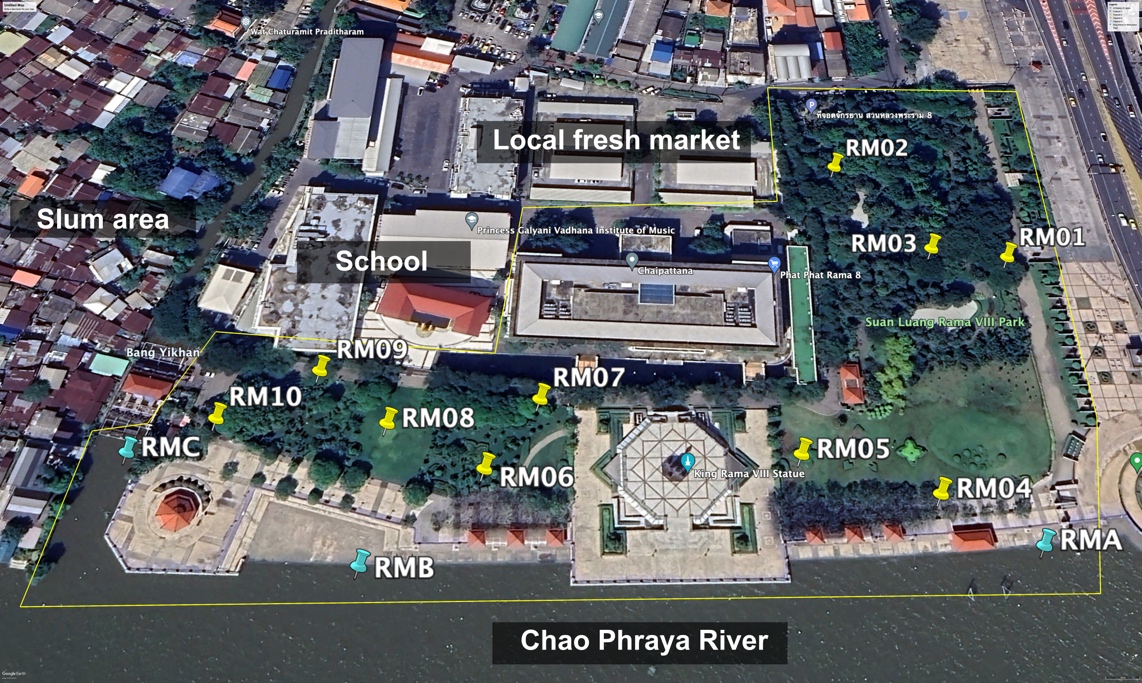 |
| (H) | 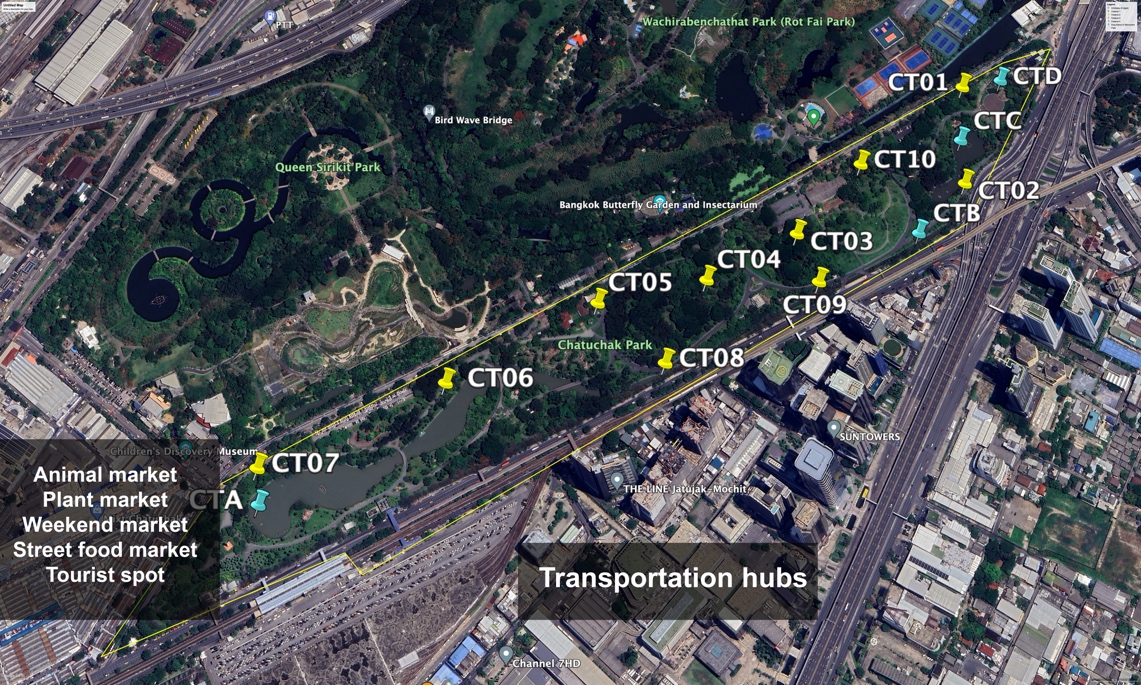 |
| (I) | 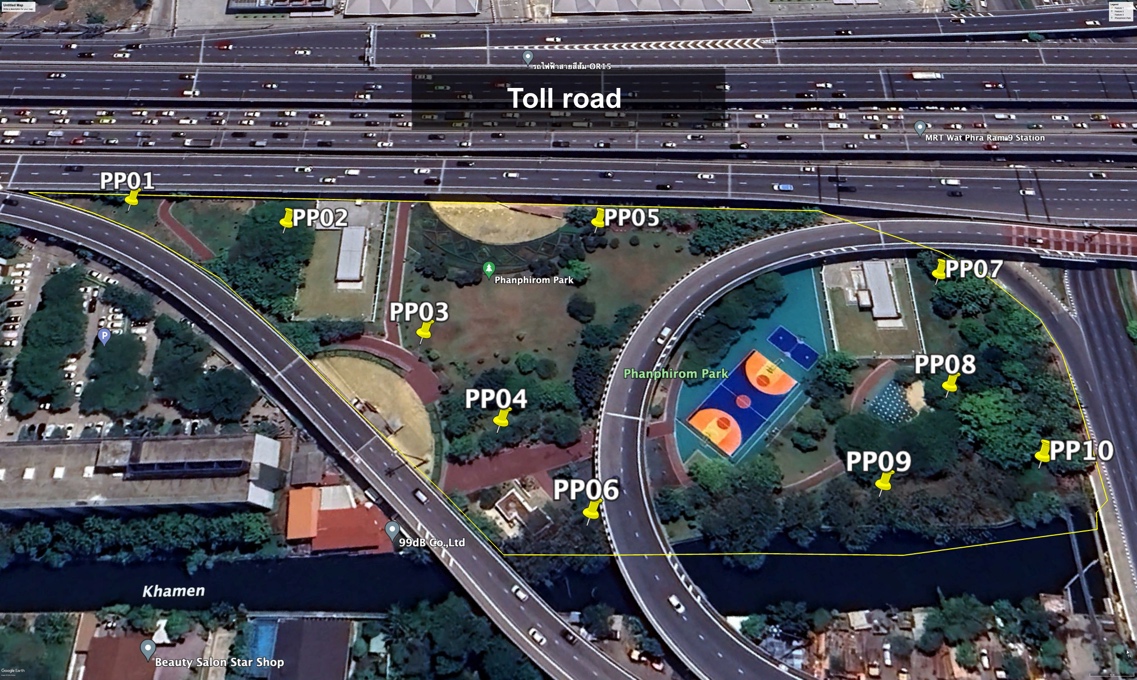 |
| (J) | 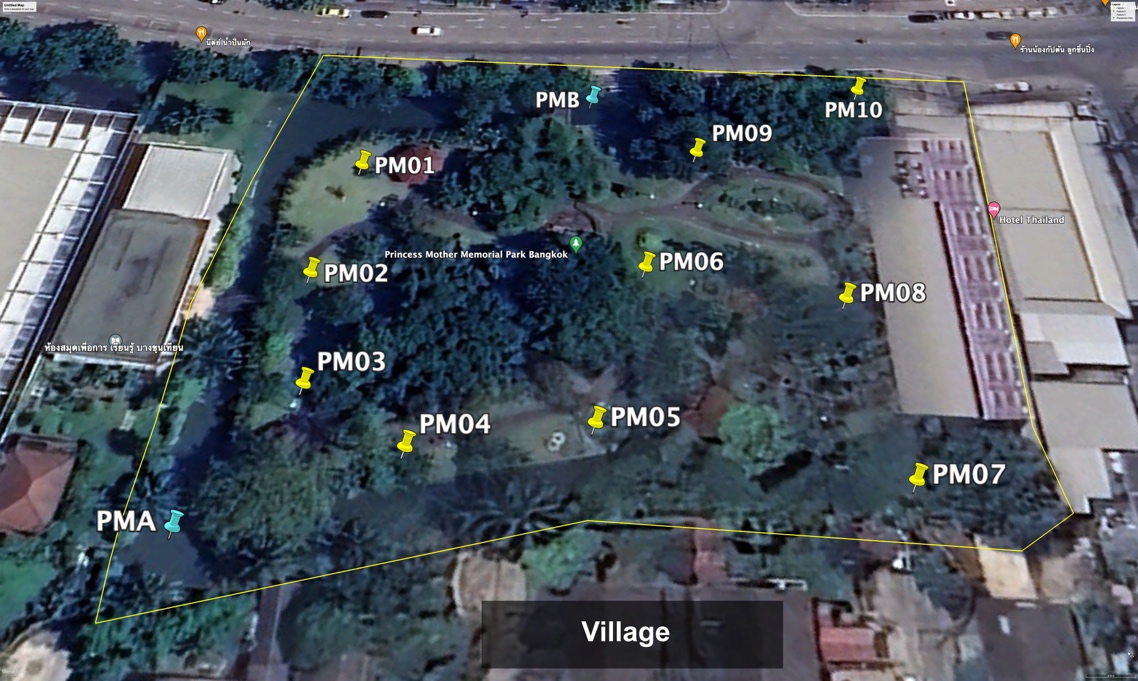 |
| (K) | 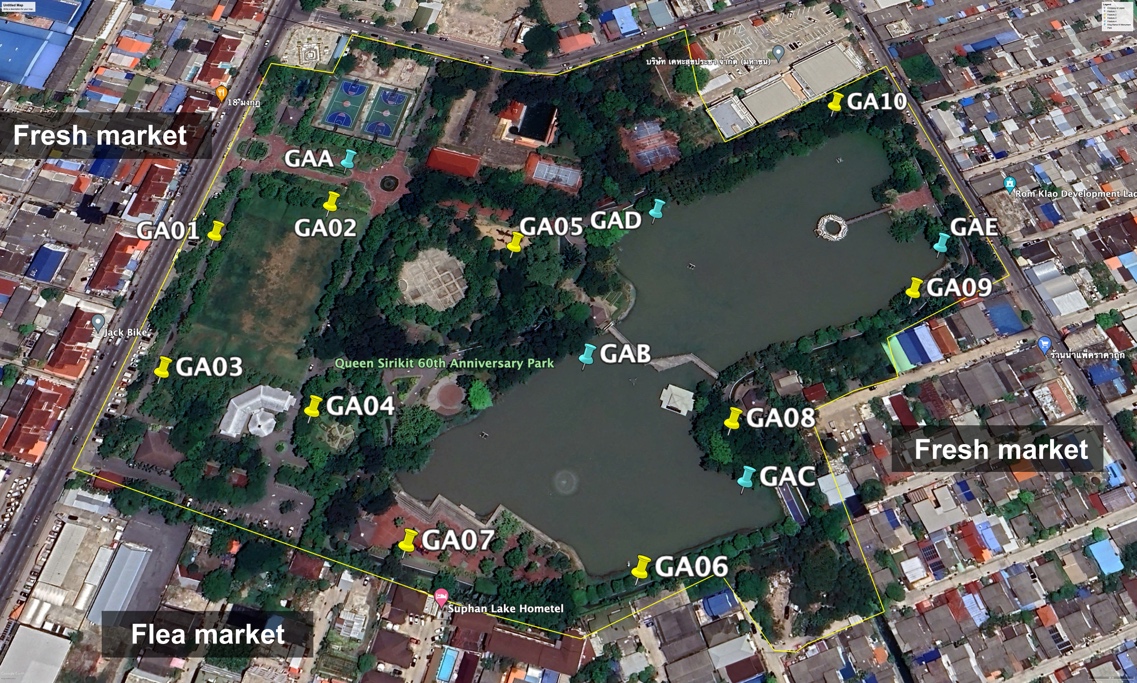 |
| (L) | 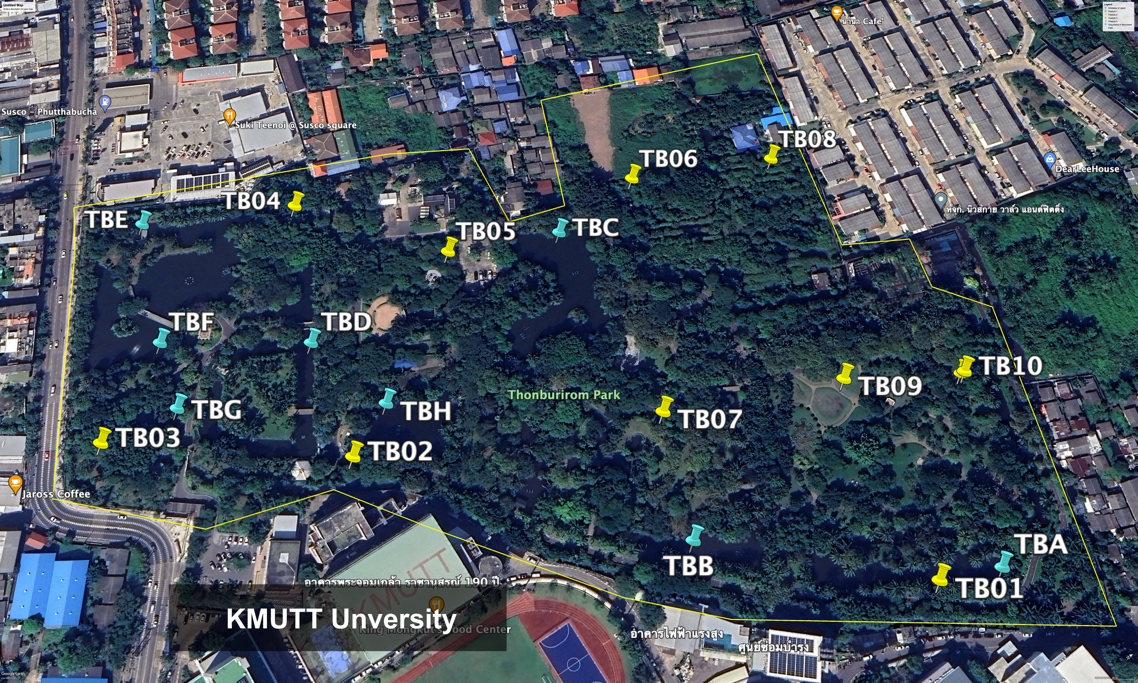 |
