# Supplementary material for: Environmental Yeast Abundance and Diversity Assessment in Recreation Areas of Bangkok, Thailand
Source: Environ Microbiol Rep. 2025 Oct 21;17(5):e70212. doi: 10.1111/1758-2229.70212 (PMC12539370; doi:10.1111/1758-2229.70212)
Supplement: Supplementary file 3 — Table S1: The temperature and humidity of each month in Bangkok, Thailand between 2019 and 2021 (station code: 455201). The information of temperature and relative humidity derived from Thai Meteorological Department. [file EMI4-17-e70212-s003.docx]

**Table S1** The temperature and humidity of each month in Bangkok, Thailand between 2019-2021 (station code: 455201). The information of temperature and relative humidity derived from Thai Meteorological Department.

| **Years** | **Month** | **Average temperature (°C)** | **Average relative humidity (%RH)** |
| --- | --- | --- | --- |
| **2019** | Jan | 28.9 | 68 |
|  | Feb | 30.5 | 74 |
|  | Mar | 31.6 | 75 |
|  | Apr | 32.5 | 73 |
|  | May | 32.7 | 71 |
|  | Jun | 31.9 | 77 |
|  | Jul | 30.5 | 74 |
|  | Aug | 30.8 | 77 |
|  | Sep | 30.5 | 81 |
|  | Oct | 30.8 | 75 |
|  | Nov | 29.8 | 65 |
|  | Dec * | 26.7 | 64 |
| **2020** | Jan * | 30.3 | 71 |
|  | Feb * | 31 | 70 |
|  | Mar | 31.8 | 75 |
|  | Apr | 31.9 | 72 |
|  | May | 31.7 | 71 |
|  | Jun | 31.2 | 76 |
|  | Jul | 31.1 | 75 |
|  | Aug | 31.1 | 76 |
|  | Sep | 31 | 78 |
|  | Oct | 28.9 | 82 |
|  | Nov | 30 | 67 |
|  | Dec * | 26.9 | 63 |
| **2021** | Jan * | 26.1 | 60 |
|  | Feb * | 28.2 | 66 |
|  | Mar * | 31.3 | 74 |
|  | Apr | 31.3 | 76 |
|  | May | 30.9 | 73 |
|  | Jun | 31.6 | 71 |
|  | Jul | 31.7 | 76 |
|  | Aug | 31.3 | 77 |
|  | Sep | 30.5 | 82 |
|  | Oct | 29.6 | 80 |
|  | Nov | 29.3 | 72 |
|  | Dec | 27.2 | 61 |
| * Yellow-shaded cells: the sampling period in this study | | | |
