# Supplementary material for: Environmental Yeast Abundance and Diversity Assessment in Recreation Areas of Bangkok, Thailand
Source: Environ Microbiol Rep. 2025 Oct 21;17(5):e70212. doi: 10.1111/1758-2229.70212 (PMC12539370; doi:10.1111/1758-2229.70212)
Supplement: Supplementary file 4 — Table S2: The global positioning system (GPS) coordination of sites determined using a Handy GPS application (free trial version, Binary Earth). [file EMI4-17-e70212-s002.docx]

**Table S2** The global positioning system (GPS) coordination of sites determined using a Handy GPS application (free trial version, Binary Earth)

| **Santiphap Park (ST)**  **(GPS, DMS) 13°45'47.34" N 100°32'29.36" E** | | | | | | | |
| --- | --- | --- | --- | --- | --- | --- | --- |
| Sources | Site name | GPS coordinates | | | | | |
|  |  | N | E | Accuracy | | Altitude | |
| Water | A | 13°45'46.28" N | 100°32'29.26" E | +/- 5 m | | 6 m | |
|  | B | 13°45'41.44" N | 100°32'27.56" E | +/- 5 m | | 6 m | |
|  | C | 13°45'37.55" N | 100°32'24.61" E | +/- 7 m | | 6 m | |
| Soil | 1 | 13°45'45.82" N | 100°32'29.39" E | +/- 4 m | | 18 m | |
|  | 2 | 13°45'46.07" N | 100°32'30.49" E | +/- 8 m | | 7 m | |
|  | 3 | 13°45'44.27" N | 100°32'27.82" E | +/- 9 m | | 6 m | |
|  | 4 | 13°45'43.41" N | 100°32'28.88" E | +/- 6 m | | 6 m | |
|  | 5 | 13°45'41.67" N | 100°32'27.20" E | +/- 5 m | | 7 m | |
|  | 6 | 13°45'42.60"N | 100°32'25.70"E | +/- 9 m | | 7 m | |
|  | 7 | 13°45'37.68" N | 100°32'24.48" E | +/- 6 m | | 3 m | |
|  | 8 | 13°45'39.99" N | 100°32'25.26" E | +/- 13 m | | 6 m | |
|  | 9 | 13°45'40.67" N | 100°32'25.40" E | +/- 14 m | | 5 m | |
|  | 10 | 13°45'43.40" N | 100°32'26.41" E | +/- 13 m | | 6 m | |
| **Lumpini Park (LP)**  **(GPS, DMS) 13°43'47.68"N 100°32'28.82"E** | | | | | | | |
| Sources | Site name | GPS coordinates | | | | | |
|  |  | N | E | Accuracy | | Altitude | |
| Water | A | 13°43'51.41" N | 100°32'22.38" E | +/- 7 m | | 35 m | |
|  | B | 13°43'52.78" N | 100°32'26.53" E | +/- 5 m | | 23 m | |
|  | C | 13°43'43.47" N | 100°32'34.55" E | +/- 4 m | | 41 m | |
| Soil | 1 | 13°43'43.41" N | 100°32'26.42" E | +/- 0 | | 0 m | |
|  | 2 | 13°43'55.41" N | 100°32'33.47" E | +/- 16 m | | 43 m | |
|  | 3 | 13°43'52.87" N | 100°32'29.42" E | +/- 24 m | | 33 m | |
|  | 4 | 13°43'51.43" N | 100°32'19.35" E | +/- 4 m | | 25 m | |
|  | 5 | 13°43'57.76" N | 100°32'18.29" E | +/- 9 m | | - 4 m | |
|  | 6 | 13°44'2.35" N | 100°32'23.53" E | +/- 13 m | | 2 m | |
|  | 7 | 13°43'41.93" N | 100°32'34.67" E | +/- 4m | | 9 m | |
|  | 8 | 13°43'43.46" N | 100°32'29.85" E | +/- 5 m | | 3 m | |
|  | 9 | 13°43'45.67" N | 100°32'23.51" E | +/- 10 m | | -12 m | |
|  | 10 | 13°43'48.63" N | 100°32'20.26" E | +/- 3 m | | 29 m | |
| **Benchakitti Park (BK)**  **(GPS, DMS) 13°43'48.05"N 100°33'34.19"E** | | | | | | | |
| Sources | Site name | GPS coordinates | | | | | |
|  |  | N | E | Accuracy | | Altitude | |
| Water | A | 13°43'49.77" N | 100°33'28.94" E | +/- 9 m | | 0 m | |
| Soil | 1 | 13°43'51.76" N | 100°33'27.37" E | +/- 7 m | | 2 m | |
|  | 2 | 13°43'50.89" N | 100°33'27.34" E | +/- 8 m | | 1 m | |
|  | 3 | 13°43'47.12" N | 100°33'27.64" E | +/- 7 m | | 10 m | |
|  | 4 | 13°43'41.71" N | 100°33'28.04" E | +/- 5 m | | 11 m | |
|  | 5 | 13°43'38.44" N | 100°33'28.14" E | +/- 9 m | | 19 m | |
|  | 6 | 13°43'33.79" N | 100°33'27.55" E | +/- 4 m | | 27 m | |
|  | 7 | 13°43'33.02" N | 100°33'30.69" E | +/- 12m | | 21 m | |
|  | 8 | 13°43'34.72" N | 100°33'34.72" E | +/- 7 m | | 4 m | |
|  | 9 | 13°43'37.30" N | 100°33'35.91" E | +/- 3 m | | 25 m | |
|  | 10 | 13°43'38.30" N | 100°33'34.45" E | +/- 12 m | | 14 m | |
| **Watchirabenchathat Park (WB)**  **(GPS, DMS) 13°48'46.33"N 100°33'16.3"E** | | | | | | | |
| Sources | Site name | GPS coordinates | | | | | |
|  |  | N | E | Accuracy | | Altitude | |
| Water | A | 13°48'59.01" N | 100°33'17.17" E | +/- 9 m | | 21 m | |
|  | B | 13°48'45.43" N | 100°33'6.45" E | +/- 14 m | | -1 m | |
|  | C | 13°48'40.4"N | 100°33'10.3"E | +/- 0 | | 0 m | |
| Soil | 1 | 13°48'53.3"N | 100°33'12.4"E | +/- 22 m | | 71 m | |
|  | 2 | 13°48'56.10" N | 100°33'23.22" E | +/- 12 m | | 34 m | |
|  | 3 | 13°48'40.90" N | 100°33'23.54" E | +/- 15 m | | -2 m | |
|  | 4 | 13°48'31.00" N | 100°33'16.00" E | +/- 0 | | 0 m | |
|  | 5 | 13°48'34.98" N | 100°33'3.87" E | +/- 0 | | 0 m | |
|  | 6 | 13°48'45.43" N | 100°33'6.45" E | +/- 14 m | | -1 m | |
|  | 7 | 13°48'40.4"N | 100°33'10.3"E | +/- 0 | | 0 m | |
|  | 8 | 13°48'47.1"N | 100°33'17.8"E | +/- 12 m | | -24 m | |
|  | 9 | 13°48'56.90" N | 100°33'26.98" E | +/- 10 m | | -31 m | |
|  | 10 | 13°48'35.72" N | 100°33'18.70" E | +/- 6 m | | 4 m | |
| **SuanLuang Rama IX Park (SL)**  **(GPS, DMS) 13°40'45.09"N 100°39'48.82"E** | | | | | | | |
| Sources | Site name | GPS coordinates | | | | | |
|  |  | N | E | Accuracy | | Altitude | |
| Water | A | 13°41'15.80" N | 100°39'58.50" E | +/- 8 m | | 0 m | |
|  | B | 13°41'15.08" N | 100°39'58.82" E | +/- 3 m | | 3 m | |
|  | C | 13°41'15.43" N | 100°39'40.60" E | +/- 4 m | | 16 m | |
| Soil | 1 | 13°41'15.40" N | 100°39'59.03" E | +/- 4 m | | 9 m | |
|  | 2 | 13°41'16.94" N | 100°39'49.57" E | +/- 6 m | | 10 m | |
|  | 3 | 13°41'21.53" N | 100°39'49.64" E | +/- 22 m | | 10 m | |
|  | 4 | 13°41'21.24" N | 100°39'43.87" E | +/- 4 m | | 20 m | |
|  | 5 | 13°41'14.57" N | 100°39'35.86" E | +/- 19 m | | 9 m | |
|  | 6 | 13°41'7.44" N | 100°39'45.71" E | +/- 6 m | | 31 m | |
|  | 7 | 13°41'1.08" N | 100°39'43.11" E | +/- 27 m | | 40 m | |
|  | 8 | 13°40'52.11" N | 100°39'40.92" E | +/- 16 m | | -11 m | |
|  | 9 | 13°40'55.71" N | 100°39'36.26" E | +/- 33 m | | 0 m | |
|  | 10 | 13°40'49.31" N | 100°39'36.57" E | +/- 20 m | | 9 m | |
| **Chaloem Phrakiat 80 Phansa Park (CP)**  **(GPS, DMS) 13°46'19.83"N 100°28'6.41"E** | | | | | | | |
| Sources | Site name | GPS coordinates | | | | | |
|  |  | N | E | | Accuracy | | Altitude |
| Water | A | 13°46'21.64"N | 100°28'9.44"E | | +/- 9 m | | 21 m |
|  | B | 13°46'24.98"N | 100°28'12.87"E | | +/- 8 m | | 12 m |
|  | C | 13°46'24.09"N | 100°28'8.36"E | | +/- 9 m | | -7 m |
| Soil | 1 | 13°46'19.83"N | 100°28'6.41"E | | +/- 5 m | | 11 m |
|  | 2 | 13°46'21.64"N | 100°28'9.44"E | | +/- 9 m | | 21 m |
|  | 3 | 13°46'23.51"N | 100°28'10.85"E | | +/- 17 m | | -5 m |
|  | 4 | 13°46'23.00"N | 100°28'13.95"E | | +/- 91 m | | 81 m |
|  | 5 | 13°46'25.76"N | 100°28'13.09"E | | +/- 11 m | | 20 m |
|  | 6 | 13°46'26.64"N | 100°28'12.39"E | | +/- 7 m | | 25 m |
|  | 7 | 13°46'25.95"N | 100°28'10.54"E | | +/- 13 m | | 4 m |
|  | 8 | 13°46'23.87"N | 100°28'8.31"E | | +/- 10 m | | 6 m |
|  | 9 | 13°46'23.00"N | 100°28'6.77"E | | +/- 28 m | | 2 m |
|  | 10 | 13°46'20.47"N | 100°28'5.60"E | | +/- 10 m | | 13 m |
| **Rama VIII Park (RM)**  **(GPS, DMS) 13°46'9.48"N 100°29'41.92"E** | | | | | | | |
| Sources | Site name | GPS coordinates | | | | | |
|  |  | N | E | Accuracy | | Altitude | |
| water | A | 13°46'8.58" N | 100°29'44.52" E | +/- 2 m | | 7 m | |
|  | B | 13°46'2.62" N | 100°29'41.35" E | +/- 2 m | | 4 m | |
|  | C | 13°46'0.91"N | 100°29'39.09"E | +/- 2 m | | 4 m | |
| Soil | 1 | 13°46'10.54" N | 100°29'41.72" E | +/- 2 m | | 5 m | |
|  | 2 | 13°46'9.56" N | 100°29'39.62" E | +/- 4 m | | 31 m | |
|  | 3 | 13°46'9.88" N | 100°29'40.74" E | +/- 2 m | | 2 m | |
|  | 4 | 13°46'8.02" N | 100°29'43.66" E | +/- 2 m | | 6 m | |
|  | 5 | 13°46'7.03" N | 100°29'43.15" E | +/- 2 m | | 5 m | |
|  | 6 | 13°46'3.99" N | 100°29'41.70" E | +/- 3 m | | 3 m | |
|  | 7 | 13°46'5.06" N | 100°29'40.81" E | +/- 2 m | | 8 m | |
|  | 8 | 13°46'3.55" N | 100°29'40.34" E | +/- 2 m | | 7 m | |
|  | 9 | 13°46'3.25" N | 100°29'39.53" E | +/- 3 m | | 5 m | |
|  | 10 | 13°46'2.42" N | 100°29'39.30" E | +/- 3 m | | 7 m | |
| **Garden 60th Anniversary Queen Park**  **(GPS, DMS) 13°45'48.41"N 100°43'49.11"E** | | | | | | | |
| Sources | Site name | GPS coordinates | | | | | |
|  |  | N | E | Accuracy | | Altitude | |
| water | A | 13°45'51.03" N | 100°43'53.98" E | +/- 2 m | | 5 m | |
|  | B | 13°45'46.7"N | 100°43'56.5"E | +/- 0 m | | 0 m | |
|  | C | 13°45'43.84" N | 100°43'58.72" E | +/- 3 m | | 3 m | |
|  | D | 13°45'48.82" N | 100°43'58.84" E | +/- 2 m | | 7 m | |
|  | E | 13°45'46.05"N | 100°44'3.03"E | +/- 3 m | | 6 m | |
| Soil | 1 | 13°45'50.49" N | 100°43'51.50" E | +/- 4 m | | 8 m | |
|  | 2 | 13°45'50.96" N | 100°43'53.44" E | +/- 3 m | | 7 m | |
|  | 3 | 13°45'48.18" N | 100°43'49.96" E | +/- 2 m | | 5 m | |
|  | 4 | 13°45'46.88" N | 100°43'52.23" E | +/- 2 m | | 2 m | |
|  | 5 | 13°45'48.55" N | 100°43'56.24" E | +/- 2 m | | 2 m | |
|  | 6 | 13°45'42.89" N | 100°43'56.18" E | +/- 4 m | | 3 m | |
|  | 7 | 13°45'44.43" N | 100°43'53.06" E | +/- 2 m | | 6 m | |
|  | 8 | 13°45'44.47" N | 100°43'58.42" E | +/- 3 m | | 0 m | |
|  | 9 | 13°45'45.45" N | 100°44'2.35" E | +/- 3 m | | 0 m | |
|  | 10 | 13°45'49.60" N | 100°44'3.28" E | +/- 6 m | | 14 m | |
| **Thonburirom Park (TB)**  **(GPS, DMS) 13°39'7.24"N 100°29'29.06"E** | | | | | | | |
| Sources | Site name | GPS coordinates | | | | | |
|  |  | N | E | Accuracy | | Altitude | |
| water | A | 13°39'11.23"N | 100°29'35.93"E | +/- 4 m | | 20 m | |
|  | B | 13°39'7.08"N | 100°29'32.69"E | +/- 6 m | | 22 m | |
|  | C | 13°39'8.83"N | 100°29'26.25"E | +/- 3 m | | 0 m | |
|  | D | 13°39'4.00"N | 100°29'25.94"E | +/- 2 m | | -6 m | |
|  | E | 13°39'2.12"N | 100°29'21.57"E | +/- 2 m | | -1 m | |
|  | F | 13°39'1.73"N | 100°29'24.72"E | +/- 2 m | | -3 m | |
|  | G | 13°39'1.59"N | 100°29'25.26"E | +/- 3 m | | 4 m | |
|  | H | 13°39'4.09"N | 100°29'27.42"E | +/- 2 m | | 2 m | |
| Soil | 1 | 13°39'10.90"N | 100°29'35.66"E | +/- 4 m | | -4 m | |
|  | 2 | 13°39'3.97"N | 100°29'27.30"E | +/- 2 m | | 3 m | |
|  | 3 | 13°39'2.82"N | 100°29'26.56"E | +/- 6 m | | -1 m | |
|  | 4 | 13°39'4.89"N | 100°29'23.15"E | +/- 3 m | | -4 m | |
|  | 5 | 13°39'6.79"N | 100°29'25.64"E | +/- 3 m | | -2 m | |
|  | 6 | 13°39'10.50"N | 100°29'26.25"E | +/- 2 m | | 2 m | |
|  | 7 | 13°39'8.28"N | 100°29'30.48"E | +/- 3 m | | -4 m | |
|  | 8 | 13°39'12.97"N | 100°29'27.40"E | +/- 2 m | | -1 m | |
|  | 9 | 13°39'11.26"N | 100°29'31.77"E | +/- 3 m | | 5 m | |
|  | 10 | 13°39'13.04"N | 100°29'32.84"E | +/- 4 m | | 10 m | |
|  | | | | | | | |
| **Chatuchak Park (CT)**  **(GPS, DMS) 13°48'27.75"N 100°33'12.19"E** | | | | | | | |
| Sources | Site name | GPS coordinates | | | | | |
|  |  | N | E | Accuracy | | Altitude | |
| water | A | 13°48'10.56"N | 100°33'9.49"E | +/- 2 m | | 6 m | |
|  | B | 13°48'40.06"N | 100°33'30.28"E | +/- 3 m | | 7 m | |
|  | C | 13°48'45.38"N | 100°33'29.58"E | +/- 2 m | | -1 m | |
|  | D | 13°48'50.71"N | 100°33'29.90"E | +/- 2 m | | 3 m | |
| Soil | 1 | 13°48'50.62"N | 100°33'30.19"E | +/- 4 m | | 4 m | |
|  | 2 | 13°48'44.71"N | 100°33'29.23"E | +/- 2 m | | -5 m | |
|  | 3 | 13°48'36.58"N | 100°33'25.05"E | +/- 4 m | | -4 m | |
|  | 4 | 13°48'31.37"N | 100°33'21.04"E | +/- 4 m | | -18 m | |
|  | 5 | 13°48'28.71"N | 100°33'18.98"E | +/- 6 m | | -12 m | |
|  | 6 | 13°48'18.87"N | 100°33'14.12"E | +/- 3 m | | 11 m | |
|  | 7 | 13°48'10.44"N | 100°33'9.24"E | +/- 2 m | | 8 m | |
|  | 8 | 13°48'27.68"N | 100°33'22.52"E | +/- 3 m | | 1 m | |
|  | 9 | 13°48'35.30"N | 100°33'25.09"E | +/- 3 m | | 3 m | |
|  | 10 | 13°48'40.61"N | 100°33'29.74"E | +/- 3 m | | 6 m | |
| **Phanphirom Park (PP)**  **(GPS, DMS) 13°45'5.49"N 100°33'30"E** | | | | | | | |
| Sources | Site name | GPS coordinates | | | | | |
|  |  | N | E | | Accuracy | | Altitude |
| Soil | 1 | 13°45'7.18"N | 100°35'26.99"E | | +/- 6 m | | 19 m |
|  | 2 | 13°45'6.48"N | 100°35'28.35"E | | +/- 4 m | | 9 m |
|  | 3 | 13°45'4.96"N | 100°35'29.29"E | | +/- 6 m | | 6 m |
|  | 4 | 13°45'3.96"N | 100°35'29.70"E | | +/- 7 m | | 1 m |
|  | 5 | 13°45'5.60"N | 100°35'31.14"E | | +/- 4 m | | 0 m |
|  | 6 | 13°45'2.99"N | 100°35'30.16"E | | +/- 4 m | | 20 m |
|  | 7 | 13°45'4.13"N | 100°35'33.91"E | | +/- 4 m | | 7 m |
|  | 8 | 13°45'3.11"N | 100°35'33.44"E | | +/- 3 m | | 8 m |
|  | 9 | 13°45'2.50"N | 100°35'32.49"E | | +/- 2 m | | 8 m |
|  | 10 | 13°45'2.32"N | 100°35'33.85"E | | +/- 8 m | | -7 m |
| **Princess Mother Memorial Park (PM)**  **(GPS, DMS) 13°38'42.27"N 100°25'17.06"E** | | | | | | | |
| Sources | Site name | GPS coordinates | | | | | |
|  |  | N | E | | Accuracy | | Altitude |
| water | A | 13°38'42.12"N | 100°25'15.39"E | | +/- 3 m | | -1 m |
|  | B | 13°38'42.27"N | 100°25'17.91"E | | +/- 2m | | -1 m |
| Soil | 1 | 13°38'43.22"N | 100°25'16.70"E | | +/-2 m | | 11 m |
|  | 2 | 13°38'43.24"N | 100°25'16.18"E | | +/-2 m | | 6 m |
|  | 3 | 13°38'42.91"N | 100°25'15.72"E | | +/-4 m | | -2 m |
|  | 4 | 13°38'42.43"N | 100°25'15.81"E | | +/-3 m | | -4 m |
|  | 5 | 13°38'41.92"N | 100°25'15.89"E | | +/-3 m | | 3 m |
|  | 6 | 13°38'41.55"N | 100°25'16.60"E | | +/-2 m | | -9 m |
|  | 7 | 13°38'41.47"N | 100°25'17.08"E | | +/-2 m | | 3 m |
|  | 8 | 13°38'41.57"N | 100°25'17.27"E | | +/-2 m | | 0 m |
|  | 9 | 13°38'42.27"N | 100°25'17.91"E | | +/-2 m | | -1 m |
|  | 10 | 13°38'41.99"N | 100°25'18.81"E | | +/-3 m | | -1 m |
