# Supplementary material for: Environmental Yeast Abundance and Diversity Assessment in Recreation Areas of Bangkok, Thailand
Source: Environ Microbiol Rep. 2025 Oct 21;17(5):e70212. doi: 10.1111/1758-2229.70212 (PMC12539370; doi:10.1111/1758-2229.70212)
Supplement: Supplementary file 5 — Table S3: Primers used to analyse the yeast isolations. [file EMI4-17-e70212-s006.docx]

**Table S3** Primers used to analyze the yeast isolations.

| **Target region** | **Primer name** | **Primer sequence (5’ → 3’)** | **Description** | **PCR length in base pair (bp)** | **PCR^c^ condition** | **Ref.** |
| --- | --- | --- | --- | --- | --- | --- |
| ITS region | ITS 5^a^ | 5’-GGAAGTAAAAGTCGTAACAAGG-3’ | The standard primary barcoding marker for fungi amplifying both ITS1 and ITS2 intron regions | 400-900 bp | Initial denaturation: 95°C, 5 mins  Denaturation: 96°C, 30 sec  Annealing: 55°C, 30 sec  Extension: 72°C, 30 sec  Final extension: 72°C, 5 mins | (Martin and Rygiewicz 2005; Schoch et al. 2012) |
|  | ITS 4^b^ | 5’-TCCTCCGCTTATTGATATGC-3’ |  |  |  |  |

ITS = internal transcribed spacer region

^a^ Forward primer

^b^ Reverse primer

^c^ PCR condition; temperature and duration
