# Supplementary material for: Environmental Yeast Abundance and Diversity Assessment in Recreation Areas of Bangkok, Thailand
Source: Environ Microbiol Rep. 2025 Oct 21;17(5):e70212. doi: 10.1111/1758-2229.70212 (PMC12539370; doi:10.1111/1758-2229.70212)
Supplement: Supplementary file 7 — Table S5: The species richness and diversity indices of yeast genera in different recreation areas. Specie richness = calculation for the number of observed species in each location. Shannon Diversity Index and Evenness were used to estimate the species diversity. [file EMI4-17-e70212-s004.docx]

**Table S5** The species richness and diversity indices of yeast genera in different recreation areas. Specie richness = calculation for the number of observed species in each location. Shannon Diversity Index and Evenness were used to estimate the species diversity.

| **Parks** | **Species richness (S)** | **Shannon Diversity Index (H')** | **H' Max** | **Evenness (J)**  (H/Hmax)  =(H/(ln(richness))) | **Abundance** |
| --- | --- | --- | --- | --- | --- |
| Chatuchak Park | 6 | 1.395 | 1.792 | 0.779 | 45 |
| Garden 60th Anniversary Queen Park | 12 | 1.948 | 2.485 | 0.784 | 43 |
| Wachirabenchathat Park | 2 | 0.410 | 0.693 | 0.592 | 14 |
| Benchakitti Park | 3 | 1.058 | 1.099 | 0.963 | 13 |
| Lumphini Park | 3 | 0.600 | 1.099 | 0.546 | 11 |
| Suan Luang Rama IX Park | 2 | 0.562 | 0.693 | 0.811 | 4 |
| Rama VIII Park | 10 | 1.952 | 2.303 | 0.848 | 23 |
| Chaloem Prekiat 80 Phansa Park | 4 | 1.171 | 1.386 | 0.845 | 14 |
| Thonburirom Park | 7 | 1.748 | 1.946 | 0.898 | 12 |
| Santiphap Park | 1 | 0.000 | 0.000 | 0.000 | 2 |
| Phanphirom Park | 4 | 1.168 | 1.386 | 0.843 | 10 |
| Princess Mother Memorial Park | 4 | 1.074 | 1.386 | 0.774 | 8 |
