# Supplementary material for: Environmental Yeast Abundance and Diversity Assessment in Recreation Areas of Bangkok, Thailand
Source: Environ Microbiol Rep. 2025 Oct 21;17(5):e70212. doi: 10.1111/1758-2229.70212 (PMC12539370; doi:10.1111/1758-2229.70212)
Supplement: Supplementary file 8 — Table S6: The statistical results of one‐way ANOVA using STATA analysis. The park codes were assigned as; 1 = Santiphap Park (ST), 2 = Lumphini Park (LP), 3 = Benchakitti Park (BK), 4 = Wachirabenchathat Park (WB), 5 = Suan Luang Rama IX Park (SL), 6 = Chaloem Prekiat 80 Phansa Park (CP), 7 = Rama VIII Park (RM), 8 = Garden 60th Anniversary Queen Park (GA), 9 = Thonburirom Park (TB), 10 = Chatuchak Park (CT), 11 = Princess Mother Memorial Park (PM), and 12 = Phanphirom Park (PP). [file EMI4-17-e70212-s005.docx]

**Table S6** The statistical results of one-way ANOVA using STATA analysis. The park codes were assigned as; 1=Santiphap Park (ST), 2=Lumphini Park (LP), 3=Benchakitti Park (BK), 4=Wachirabenchathat Park (WB), 5=Suan Luang Rama IX Park (SL), 6=Chaloem Prekiat 80 Phansa Park (CP), 7= Rama VIII Park (RM), 8= Garden 60th Anniversary Queen Park (GA), 9= Thonburirom Park (TB), 10= Chatuchak Park (CT), 11=Princess Mother Memorial Park (PM), and 12=Phanphirom Park (PP).

. oneway numberofitsidentify parkcode, tabulate bonferroni

| Summary of Number of ITS identify

Park code | Mean Std. Dev. Freq.

------------+------------------------------------

1 | .15384615 .5547002 13

2 | .84615385 1.675617 13

3 | 1.1818182 3.600505 11

4 | 1.0769231 2.7526211 13

5 | .30769231 .85485041 13

6 | 1.0769231 1.1875422 13

7 | 1.7692308 2.0475126 13

8 | 2.8666667 3.7581657 15

9 | .66666667 1.2833779 18

10 | 3.2142857 7.0730101 14

11 | .66666667 .98473193 12

12 | 1 2.4944383 10

------------+------------------------------------

Total | 1.2594937 3.0014442 158
